# Changes in chromatin accessibility ensure robust cell cycle exit in terminally differentiated cells

**DOI:** 10.1371/journal.pbio.3000378

**Published:** 2019-09-03

**Authors:** Yiqin Ma, Daniel J. McKay, Laura Buttitta

**Affiliations:** 1 Department of Molecular Cellular and Developmental Biology, University of Michigan, Ann Arbor, Michigan, United States of America; 2 Department of Biology, Department of Genetics, Integrative Program for Biological and Genome Sciences, University of North Carolina at Chapel Hill, Chapel Hill, North Carolina, United States of America; UCLA, UNITED STATES

## Abstract

During terminal differentiation, most cells exit the cell cycle and enter into a prolonged or permanent G0 in which they are refractory to mitogenic signals. Entry into G0 is usually initiated through the repression of cell cycle gene expression by formation of a transcriptional repressor complex called dimerization partner (DP), retinoblastoma (RB)-like, E2F and MuvB (DREAM). However, when DREAM repressive function is compromised during terminal differentiation, additional unknown mechanisms act to stably repress cycling and ensure robust cell cycle exit. Here, we provide evidence that developmentally programmed, temporal changes in chromatin accessibility at a small subset of critical cell cycle genes act to enforce cell cycle exit during terminal differentiation in the *Drosophila melanogaster* wing. We show that during terminal differentiation, chromatin closes at a set of pupal wing enhancers for the key rate-limiting cell cycle regulators *Cyclin E* (*cycE*), *E2F transcription factor 1* (*e2f1*), and *string* (*stg*). This closing coincides with wing cells entering a robust postmitotic state that is strongly refractory to cell cycle reactivation, and the regions that close contain known binding sites for effectors of mitogenic signaling pathways such as Yorkie and Notch. When cell cycle exit is genetically disrupted, chromatin accessibility at cell cycle genes remains unaffected, and the closing of distal enhancers at *cycE*, *e2f1*, and *stg* proceeds independent of the cell cycling status. Instead, disruption of cell cycle exit leads to changes in accessibility and expression of a subset of hormone-induced transcription factors involved in the progression of terminal differentiation. Our results uncover a mechanism that acts as a cell cycle–independent timer to limit the response to mitogenic signaling and aberrant cycling in terminally differentiating tissues. In addition, we provide a new molecular description of the cross talk between cell cycle exit and terminal differentiation during metamorphosis.

## Abstract 2

The longer a cell remains in G0, the more refractory it becomes to re-entering the cell cycle. This study shows that in terminally differentiated cells in vivo, regulatory elements at genes encoding just three key cell cycle regulators (cycE, e2f1 and stg) become inaccessible, limiting their aberrant activation and maintaining a prolonged, robust G0.

## Introduction

The majority of cells in mature multicellular organisms spend most of their existence in nonproliferating states, often referred to as cellular quiescence or the G0 phase [1]. Substantial progress has been made on understanding how developmental signaling pathways interface with the cell cycle machinery to control tissue growth and proliferation [2,3]. Yet we understand very little about why some cell types can enter a more flexible G0 state and retain the ability to reenter the cell cycle in response to mitogens, whereas others become permanently postmitotic and refractory to mitogenic signaling. Robust and synchronous silencing of cell cycle gene expression is critical to the proper timing of cell cycle exit and the maintenance of a postmitotic state. Yet the molecular details of how this silencing is initiated and maintained in maturing tissues remain unresolved. This impacts a wide range of biological questions, as the proper control of G0 is critical during development and tissue regeneration but becomes disrupted in cancer.

The transition from proliferation to G0 is accompanied by a functional switch in the master regulators of the cell cycle program, such as E2F transcription factor (E2F) complexes, leading to global down-regulation of cell cycle gene transcription [4–6]. In proliferating cells, activating E2F family members (dE2F1 in *Drosophila*) bind with the dimerization partner (DP) at promoter proximal E2F binding motifs at hundreds of cell cycle genes, including cyclins, cyclin-dependent kinases (Cdks), replication proteins, and mitotic regulators, to promote their expression. Upon entry into G0, silencing through these same binding sites occurs via the formation of a transcriptional repressor complex called DP, retinoblastoma (RB)-like, E2F and MuvB (DREAM). DREAM complexes are conserved from *Caenorhabditis elegans* to humans, and in *Drosophila*, DREAM (termed dREAM) consists of the E2F binding partner DP, Retinoblastoma-family protein Rbf1 or Rbf2, the repressive E2F transcription factor family member dE2F2, chromatin assembly factor 1, p55 subunit (p55/CAF1), Myb oncogene-like protein (Myb), and Myb-interacting proteins [4,6]. Formation of dREAM is promoted by the accumulation of hypophosphorylated or unphosphorylated RB through the inhibition of cyclin/Cdk. Therefore, it can be induced through developmental activation of Cdk inhibitors, developmental down-regulation of the expression and production of cyclins and cdks, or the up-regulation of cyclin destruction through the anaphase-promoting complex/cyclosome (APC/C) [7–10].

Although dREAM plays an important role in the repression of cell cycle genes in G0, key aspects of cell cycle exit in vivo are still not understood. For example, in some contexts of differentiation, cells eventually arrest and differentiate even in the presence of constitutive high E2F or cyclin/Cdk activity [11–15]. We and others have found that this is in part due to cooperative roles for RBs, cyclin kinase inhibitors, and the APC/C. Double and triple combinations of alleles altering these pathways cooperate to further delay cell cycle exit, but they fail to abrogate it completely, suggesting these pathways act in addition to still-unknown developmental mechanisms [8,9,16–19]. These redundant mechanisms make cell cycle exit upon terminal differentiation more robust than other states of G0, such as reversible quiescence.

We and others have also observed that the longer a terminally differentiated cell remains in G0, the more refractory it becomes to reentering the cell cycle, even in the presence of high E2F or cyclin/Cdk activity [20]. This has been termed “deep” or “robust” G0 [9,21]. The molecular basis of robust G0 and how it differs from temporary or “flexible” G0 states remains unknown. One model for how terminally differentiated cells become resistant to strong proliferation signals involves a chromatin lockdown mechanism, in which chromatin compaction or repressive modifications act globally to silence cell cycle gene expression and promote robust cell cycle exit. For example, DREAM complexes can recruit chromatin modifiers to add repressive histone modifications at E2F-dependent cell cycle genes such as H3K27 trimethylation (H3K27Me3) or H3K9 trimethylation (H3K9Me3), which in turn recruit repressive heterochromatin binding proteins such as the Polycomb repressive complex 1 (PRC1) or heterochromatin protein 1 (HP1) for long-term silencing of cell cycle genes [22,23]. Another model posits that cell cycle genes become recruited to the nuclear periphery to be sequestered in repressive nuclear lamina-associated domains (LADs) [24]. We directly tested these models in the terminally differentiating *Drosophila* wing and found cell cycle exit occurs despite disruption of heterochromatin-dependent gene silencing [25] and without obvious sequestration or recruitment of cell cycle genes to heterochromatin compartments or the nuclear lamina. This suggests developmentally controlled cell cycle exit in *Drosophila* uses additional mechanisms to ensure a robust G0.

Cell cycle exit in the *Drosophila* wing occurs during metamorphosis and is tied to pulses of the hormone ecdysone that induce downstream transcription factors to modulate cell cycle gene expression [26]. We recently showed that transcription factors downstream of ecdysone signaling play a critical role in promoting sequential changes in chromatin accessibility to promote wing differentiation [27]. This suggested to us that changes in chromatin accessibility during metamorphosis could contribute to the regulation of cell cycle genes to coordinate cell cycle exit with differentiation. To examine this, we characterized the transcriptome and genome-wide chromatin accessibility landscape of the *Drosophila* wing during metamorphosis through RNA sequencing (RNA-seq) and formaldehyde-assisted isolation of regulatory elements (FAIRE) sequencing (FAIRE-seq) over six developmental time points. We show that during wing differentiation, chromatin accessibility and gene expression changes are coordinated with the transition from a proliferating to a postmitotic state. This includes the closing of specific regulatory elements at a subset of critical “master” cell cycle regulators during G0. Moreover, we have uncoupled differentiation from cell cycle exit, revealing that the closing of pupa wing enhancers at these cell cycle master regulator genes is developmentally programmed and occurs independent of E2F activity or cell cycling status, coincident with robust G0. We propose that the developmentally programmed closing of regulatory elements at a subset of key cell cycle genes is the molecular mechanism underlying robust cell cycle exit in vivo.

## Results

### Chromatin accessibility and gene expression are temporally dynamic during wing metamorphosis

During metamorphosis, wings undergo morphogenetic changes coordinated with cell cycle alterations, loss of regeneration capacity, and activation of a wing terminal differentiation program [28–30]. These events are temporally coordinated by systemic hormone pulses, which trigger metamorphosis and drive its progression, leading to coordinated morphogenesis and differentiation of organs [31,32]. Although the hormone pulses are systemic, through a combination of direct and indirect regulation, they result in activation of unique gene expression programs in different tissues [27,33–35]. For the wing, major events during metamorphosis include the following: eversion coordinated with a temporary cell cycle arrest in G2 and pupa cuticle formation; elongation and apposition of dorsal and ventral surfaces, coordinated with a relatively synchronized final cell cycle and vein refinement; and finally, permanent cell cycle arrest, which precedes wing hair formation and deposition of adult cuticle [26,36–39]. Underlying these processes are temporally coordinated changes in gene expression. We and others have examined the dramatic gene expression changes in the wing during metamorphosis [37,40]. To identify the global landscape of potential regulatory elements driving these gene expression changes, we carried out FAIRE-seq in parallel with RNA-seq in a time course of wild-type *Drosophila* wings from the late wandering third instar stage when wing cells are proliferating to 44 h after puparium formation (APF), when wing cells are postmitotic and begin to deposit adult cuticle.

**Fig 1:**
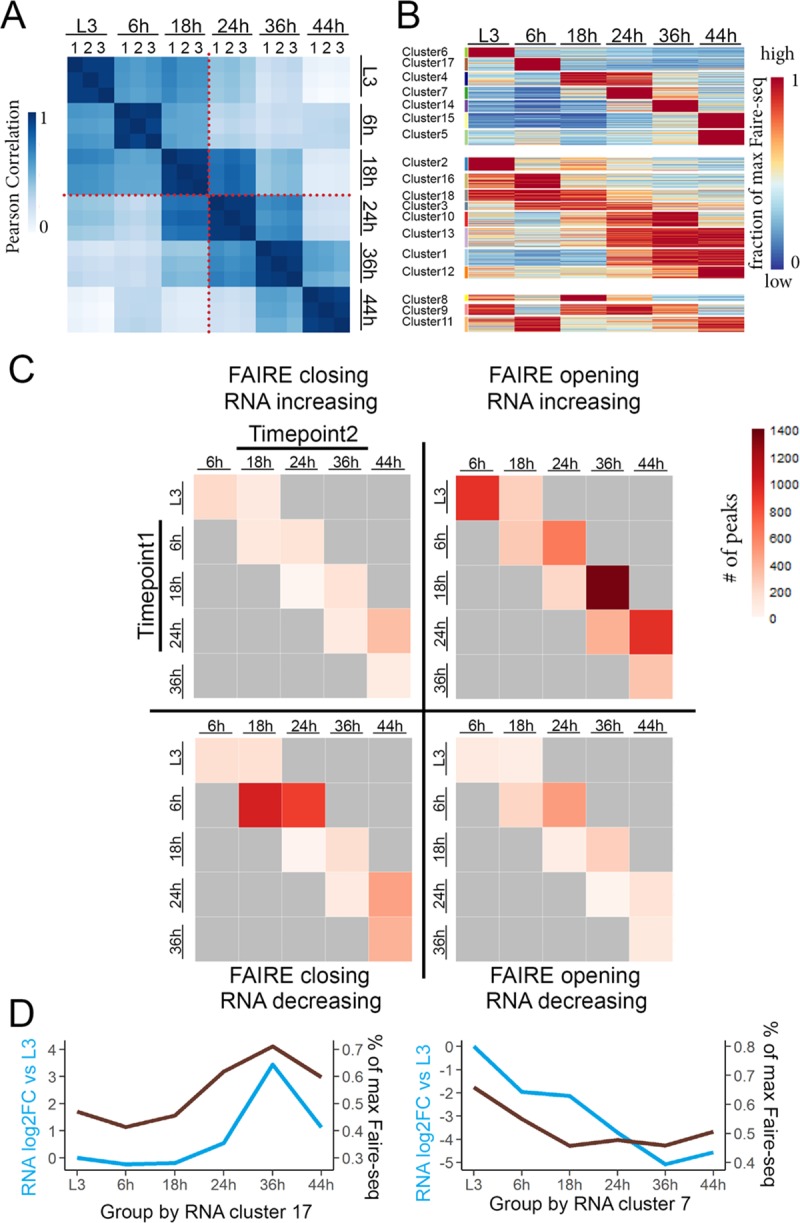
Dynamics of chromatin accessibility and gene expression are correlated during metamorphosis. (A) Open chromatin regions (peaks) in wings were identified by FAIRE-seq on time points prior to metamorphosis (L3) and during pupal stages 6 h, 18 h, 24 h, 36 h, and 44 h APF. Heatmaps of Pearson correlation coefficients for each replicate across this time course reveal differences between the proliferative and postmitotic stages (red dotted line). (B) Dynamic open chromatin peaks were organized into 18 k-means clusters, displayed as a heatmap representing the fraction of the maximum FAIRE rpkm value. (C, D) Most chromatin accessibility changes are associated with gene activation rather than repression during metamorphosis. (C) We assigned dynamic FAIRE peaks to the nearest expressed gene and correlated peak changes (opening or closing) with observed gene expression changes (increasing or decreasing) measured by RNA-seq at each subsequent time point. This revealed four classes of FAIRE peak/RNA expression correlations: opening/increasing consistent with gene activation, closing/decreasing consistent with loss of activation, opening/decreasing consistent with binding of a repressor, and closing/activation consistent with a loss of repression. We show the number of dynamic FAIRE peaks that fall into each quadrant for comparisons at time point 1 (t1) (y-axis) and time point 2 (t2) (x-axis). (D) Genes were clustered based on RNA expression patterns across metamorphosis. Two clusters showing a high positive correlation between RNA signal (average log2 fold change from L3) and accessibility of their assigned FAIRE peaks (average maximum FAIRE rpkm value) are shown. The full dataset correlating RNA expression with accessibility of their assigned FAIRE peaks for all clusters is provided in the Supporting information. The underlying data in Fig 1A–1D can be found within S1 Data. APF, after puparium formation; Cdk, cyclin-dependent kinase; FAIRE, formaldehyde-assisted isolation of regulatory elements; FAIRE-seq, FAIRE sequencing; RNA-seq, RNA sequencing; rpkm, reads per kilobase, per million mapped reads.

We identified a total of 20,329 high-confidence open chromatin regions (peaks). We first compared the similarity of open chromatin profiles across our wing developmental time course by examining Pearson correlation coefficients. The open chromatin landscape is gradually changing during metamorphosis, and early proliferative stages are clearly distinct from postmitotic stages in chromatin accessibility (Fig 1A). By calculating the fold change in peak accessibility between stages, we found that only 5,516 peaks (27%) are static and exhibit <2-fold changes between any two time points. The remaining 14,813 peaks (73%) appear developmentally dynamic, exhibiting >2-fold changes between two or more time points. To visualize peak accessibility dynamics during metamorphosis, we divided the reads per kilobase, per million mapped reads (rpkm) value of each FAIRE peak by its maximum rpkm value for each of the six time points and then plotted the fraction in the form of a heatmap (Fig 1B). To distinguish different dynamic patterns, we separated the peaks into 18 k-means clusters. We found that dynamic peaks could be divided into three broad categories: a temporally sharp category that transiently opens at only one stage, a temporally broad category that remains accessible for several sequential stages, and a category of peaks that oscillate during metamorphosis.

Consistent with previous work, our parallel RNA-seq revealed dynamic expression changes for over 6,000 genes (over 35% of the genome) during wing metamorphosis. For comparison to the FAIRE-seq clusters, we also clustered genes based on RNA expression into 18 k-means RNA-seq clusters (S1A Fig). Clustering based on gene expression identified groups of genes that are functionally related and temporally coordinated. For example, RNA cluster 4 contains genes highly expressed at 6 h, which are enriched for genes involved in wing pupal cuticle development (S1B Fig), whereas RNA clusters 7 and 10 coordinately decrease expression after 18 h and are highly enriched for cell cycle genes. This is consistent with our previous work showing that cell cycle gene expression plummets by 24 h when cells transition to a postmitotic state [26,40].

To more easily visualize the temporal dynamics of peaks, we next compared dynamic peaks between adjacent stages to define them as opening or closing compared with the previous stage (Fig 1C and 1D, S2 Fig). The time point with the most dynamic changes is 6 h, and the second-most dynamic is 24 h. Both of these stages are associated with cell cycle arrests. We previously showed that at 6 h, wings undergo a temporary G2 arrest induced by high levels of the transcription factor Broad suppressing the critical G2-M regulator cell division cycle 25c (cdc25c) or string. This synchronizes the subsequent final cell cycle [26]. At 24 h, cells in the wing finish the final cell cycle in a relatively synchronized manner and enter into a postmitotic G0 state [41,42]. This suggested to us that a developmentally controlled program of coordinated chromatin accessibility could link changes in the cell cycle with differentiation during metamorphosis.

To correlate chromatin accessibility changes with gene expression changes, we assigned dynamic and static FAIRE peaks to the nearest transcription start site (TSS) (S2 Fig). Dynamic and static FAIRE peaks exhibit similar distributions, with most of them located in introns, intergenic regions, and promoter proximal regions. This is consistent with previous work showing that FAIRE-seq enriches for DNA regulatory elements [43]. Both dynamic and static peaks were most highly enriched at locations near (1–5 kb) their assigned TSS. However dynamic peaks were also more likely to be located farther from TSSs (5–10 kb) than static peaks, which were more likely to be promoter proximal (within 0.5 kb from the assigned TSS) (S2 Fig). Similar to other studies using FAIRE in *Drosophila*, we find most developmentally dynamic putative regulatory elements for the wing are located within introns, especially the first intron and 1–5 kb upstream of the TSS. This is also consistent with the locations of *Drosophila* enhancers identified using a functional accessibility-independent approach, self-transcribing active regulatory region sequencing (Starr-Seq) [44].

### Dynamic chromatin is mostly correlated with gene activation

Open chromatin sites often correspond to gene regulatory elements such as transcriptional enhancers, which can activate or repress gene expression. To determine whether FAIRE peak dynamics correlate positively or negatively with gene expression changes, we assigned each FAIRE peak to its nearest gene and carried out pairwise comparisons between each stage and its next two sequential stages for >2-fold changes in chromatin accessibility correlated with >2-fold expression changes using our RNA-seq data (S3 Fig). When we plot peak accessibility change versus assigned gene expression changes, we generate four quadrants: FAIRE peaks opening with corresponding gene expression increasing, consistent with an activation function; FAIRE peaks opening with gene expression decreasing, consistent with a repressive function; FAIRE peaks closing with gene expression increasing, consistent with the loss of a repressor binding; and FAIRE peaks closing and gene expression decreasing, consistent with the loss of an activator binding (Fig 1C).

We observed that the majority of dynamic FAIRE peaks fall into the category of peaks opening with the corresponding gene expression increasing. This suggests that the majority of gene expression changes in the differentiating wing are driven by transcriptional activators gaining access to their binding sites. The second-largest category of FAIRE peaks close and are associated with loss of expression. This suggests that loss of access to transcriptional activators also plays a major role in gene repression during terminal differentiation.

We next examined the correlation between gene expression and chromatin accessibility changes during our time course (Fig 1D, S4 and S5 Figs). We plotted the trajectory of gene expression based upon RNA-seq for 18 coregulated gene clusters and overlaid the average changes in FAIRE peaks assigned to the genes within each RNA cluster (Fig 1D, S4 Fig). We also performed a reciprocal analysis using 18 gene clusters based upon coregulated FAIRE peaks and overlaid average gene expression changes from RNA-seq (S5 Fig). We found that for several clusters, the temporal changes in RNA and accessibility by FAIRE-seq are well correlated (Fig 1C). Together, our results suggest that most of the dynamic regulatory elements during fly wing metamorphosis are associated with gene activation.

### Opening the wing differentiation program during metamorphosis

**Fig 2:**
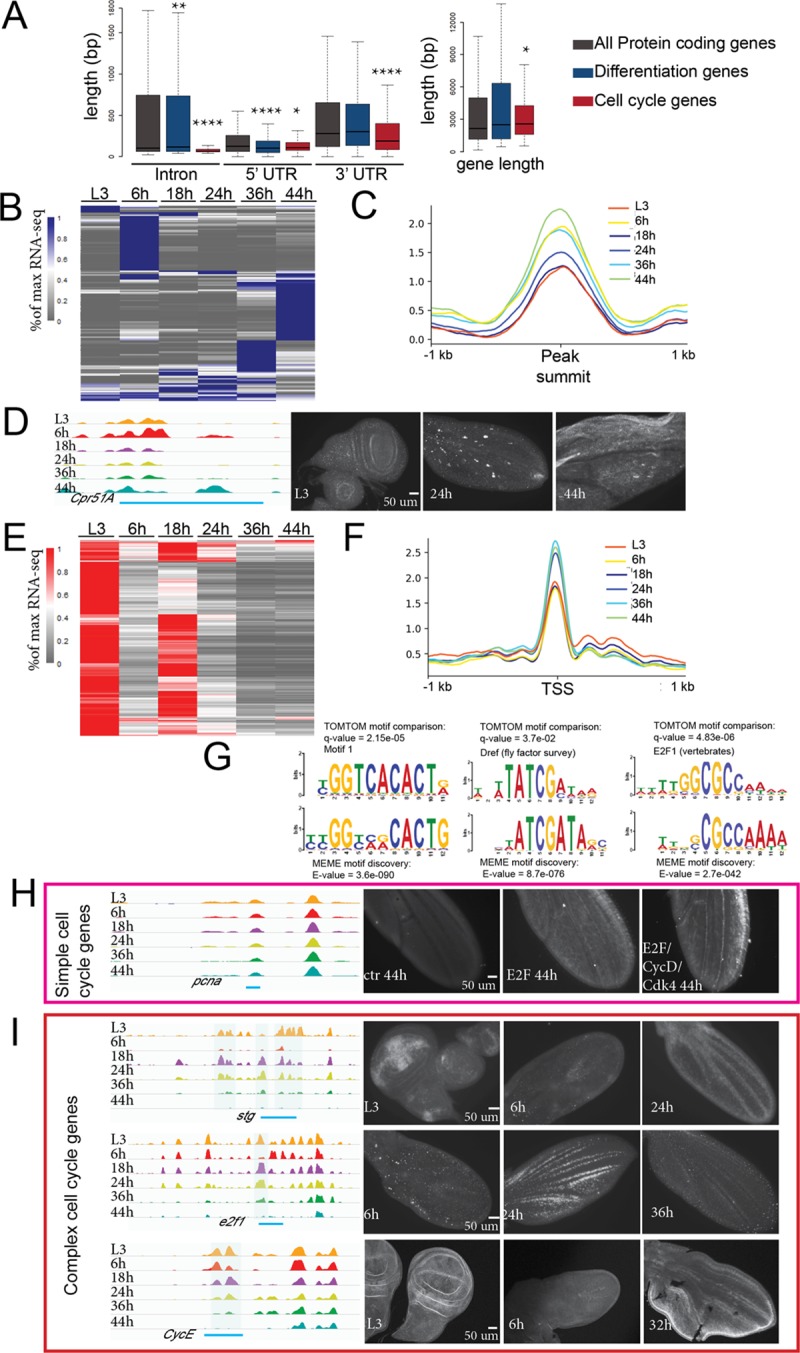
Temporal regulation of the wing differentiation program and cell cycle changes. (A) The length (in bp) of introns, 5′ UTRs, and 3′ UTRs (left) and genes (right) for all protein coding genes, wing terminal differentiation genes, and cell cycle genes is shown. The majority of FAIRE peaks occur within introns (S2 Fig). Most cell cycle genes have a compact structure with few, short introns, whereas differentiation genes contain large introns, providing potential dynamic regulatory elements. (B, E) Heatmap of gene expression for differentiation genes (B) and cell cycle genes (E), plotted by the percent of maximum RNA rpkm value. Both groups of genes show dynamic expression during metamorphosis. (C, F) Line plots of average FAIRE signal of the six stages for differentiation genes (C) and cell cycle genes (F). Differentiation genes show an increase in FAIRE peak accessibility at time points when gene expression is high: 6 h (*p*-value = 0.0004088), 36 h (*p*-value = 1.36 × 10^−7^), and 44 h (*p*-value = 1.408 × 10^−12^), compared with L3, Mann-Whitney U Test). Cell cycle genes show an increase in accessibility at time points when gene expression is repressed: 24 h (*p*-value = 0.0209), 36 h (*p*-value = 1.655 × 10^−5^), and 44 h (*p*-value = 0.005469), Mann-Whitney U Test. (D) A Gal4 reporter containing the indicated (blue bar) portion of the Cpr51A regulatory region drives UAS-GFP in late wings (44 h) when the regulatory elements are accessible. (G) Motif discovery was performed on open regions for cell cycle genes using MEME and compared with known motifs using TOMTOM. Potential regulatory elements for cell cycle genes are highly enriched for E2F binding motifs, DRE promoter sequences, and the Pol II pausing-associated motif 1. (H) A GFP reporter containing the indicated regulatory element (blue bar) for the simple cell cycle gene *pcna* is silent at the postmitotic stage of 44 h but can be reactivated postmitotically when E2F or E2F + CycD/Cdk4 is expressed. (I) *stg*, *e2f1*, and *cycE* are complex cell cycle genes with large regulatory regions. Blue shading indicates regions of known regulatory sequence that exhibit dynamic accessibility. Gal4 reporters containing the indicated portions (blue bars) of their regulatory regions drive UAS-degradable GFP to capture their regulatory functions in the pupal wing. Expression correlates with accessibility for these regions. *p*-values were determined by Mann-Whitney U Test; ****< 0.0001, **< 0.01, *< 0.05. The underlying data in Fig 2A, 2B and 2E can be found within S2 Data. Cdk, cyclin-dependent kinase; Cpr51A, Cuticular protein 51A; ctr, control; CycD, Cyclin D; CycE, Cyclin E; DRE, DNA replication-related element; E2F, E2F transcription factor; FAIRE, formaldehyde-assisted isolation of regulatory elements; GFP, green fluorescent protein; MEME, Multiple Em for Motif Elicitation; PCNA, proliferating cell nuclear antigen; Pol II, RNA polymerase II; RNA-seq, RNA sequencing; rpkm, reads per kilobase of transcript, per million mapped reads; stg, string; TOMTOM, Motif Comparison Tool; UAS, upstream activation sequence.

During metamorphosis, the wing undergoes cell cycle exit, coordinated with subsequent terminal differentiation. We therefore examined several hundred genes involved in these processes (Fig 2A). A major event during wing differentiation is the formation of the adult cuticular exoskeleton. The wing cuticle is a multilayered structure, and its formation requires the proper expression of cuticle-related genes, such as enzymes involved in cuticle deposition, and zona pellucida (ZP) domain proteins, which link the apical surface of wing cells to the overlying cuticle [37]. When we examined 154 cuticle formation–associated genes, we found distinct subgroups of genes highly expressed at 6, 36, and 44 h (Fig 2B). The cuticle genes expressed at 6 h are likely to be involved in the pupa cuticle formation [36], whereas the adult cuticle program begins at 36 h and extends to 44 h and beyond. The subgroups of cuticle-related genes reaching their peak expression at different stages suggest that waves of sequential regulation during metamorphosis may drive differences in pupa cuticle versus adult cuticle composition and structure. Highly accessible FAIRE peaks found near cuticle genes (Fig 2C) are significantly more accessible at 6, 36, and 44 h, consistent with the high expression at those time points. To identify a cuticle gene regulatory element, we examined a line containing a Gal4 transgene overlapping an open chromatin region near *Cuticular protein 51A* (*Cpr51A*) driving upstream activation sequence (UAS)–green fluorescent protein (GFP) (Fig 2D). This region is highly accessible at 44 h, and with this transgene, GFP is highly expressed in almost all the wing cells at 44 h. Our results show that opening and activation of the adult cuticle program is a major feature of wing differentiation during metamorphosis.

### Repression of most cell cycle genes is established and maintained through promoter proximal regulatory elements

Cells in pupal wings exit the cell cycle at 24 h, which accompanies temporally synchronized repression of cell cycle genes. We examined the expression of approximately 300 cell cycle genes compiled from our previous analysis of cell cycle exit [9] (Fig 2E) and observed a temporary repression during the G2 arrest at 6 h, followed by reactivation at 18 h for the final cell cycle, and silencing during cell cycle exit at 24 h. We examined the chromatin accessibility profiles for 291 of these cell cycle genes and found that most of them exhibit a compact gene structure with smaller introns and relatively short intergenic upstream sequences (Fig 2A). Most FAIRE peaks associated with these genes are found to be proximal to the TSS, consistent with the previously reported distribution for functional enhancers at “housekeeping” genes [45]. Surprisingly, putative regulatory elements at the majority of cell cycle genes exhibit a moderate increase in accessibility at time points when cells are postmitotic (24–44 h) despite the temporally regulated shutoff of their associated genes at 24 h (Fig 2F).

We carried out a de novo motif discovery on the promoter proximal regions for cell cycle genes using Multiple Em for Motif Elicitation (MEME) tool (Fig 2G). The most highly enriched motifs match Motif 1, a core promoter element bound by Motif 1 binding protein (M1BP) to promote RNA Polymerase II pausing [46,47], DNA replication-related element (DRE), a core promoter/enhancer known to be associated with cell cycle genes [48], and a motif matching the binding site for the heterodimer transcription factor complex E2F/DP (found in 130 out of 540 promoter proximal regions). The increased accessibility at these motifs is similar to the increased micrococcal nuclease sensitivity found at sites in cell cycle genes bound by repressive human DREAM complexes [49]. Although increased accessibility may seem counterintuitive when coupled with gene repression, this may be consistent with a model whereby promoter proximal DREAM binding to nucleosome-free regions represses cell cycle genes by positioning nucleosomes downstream of the transcriptional start site [49].

Multiple studies have reported that depletion of Rb family members leads to derepression of cell cycle genes and defects in exiting the cell cycle. However, it has remained unclear whether Rb- or DREAM-dependent repression is required to counteract E2F activity to initiate repression of cell cycle genes, to maintain repression in cells that have already become postmitotic, or both. To investigate this, we took advantage of a proliferating cell nuclear antigen (PCNA)-GFP transcriptional reporter that includes known E2F binding sites contained within FAIRE peaks that remain accessible after cell cycle exit [50]. At 44 h, a time point when the postmitotic state has been maintained for 20 h, the reporter is silenced. To test whether this silencing can be reversed, we activated expression of the *Drosophila* activator E2F complex E2F1/DP (hereafter, E2F) or E2F + CyclinD (CycD)/Cdk4 to phosphorylate and inactivate Rbf specifically after cells have already established a flexible G0 state at 26 h (Fig 2H). Expressing either E2F or E2F + CycD/Cdk4 was able to reinduce PCNA-GFP expression in postmitotic cells, demonstrating that RB/E2F-dependent repression is required to maintain silencing of cell cycle genes in *Drosophila*.

### The accessibility of distal regulatory elements for complex cell cycle genes is dynamic

In contrast to the majority of cell cycle genes, a few key, rate-limiting cell cycle genes are controlled by long, complex regulatory elements upstream of their TSS or in long introns. For example, *cycE*, string (*stg*), and *e2f1* fall into this group (Fig 2I). We find several FAIRE peaks in regulatory regions for these genes that overlap with previously characterized functional regulatory elements [51–54]. Here, we discovered that the accessibility of these regulatory elements is temporally dynamic during metamorphosis, in a manner coordinated with the cell cycle changes. Accessibility at these elements is low during the G2 arrest at 6 h, then rises at 18 h and 24 h, and closes after 36 h. To examine whether the dynamic accessibility of these elements impacts temporal gene expression, we tested regions from the *stg*, *e2f1*, and *cycE* loci driving a Gal4/UAS-destabilized GFP (*stg*, *e2f1*) or normal GFP (*cycE*) to capture gene expression shutoff. Our GFP reporters showed dynamic expression correlated with the accessibility of the elements, which verifies some of these distal elements as pupal wing enhancers for these cell cycle genes. However, none of these enhancers individually recapitulate the normal, broad expression of these genes in the pupa wing. Our results suggest that dynamic chromatin accessibility at specific enhancers of complex cell cycle genes drives temporal expression changes during metamorphosis.

### The closing of enhancers at complex cell cycle genes is independent of cell cycle exit

**Fig 3:**
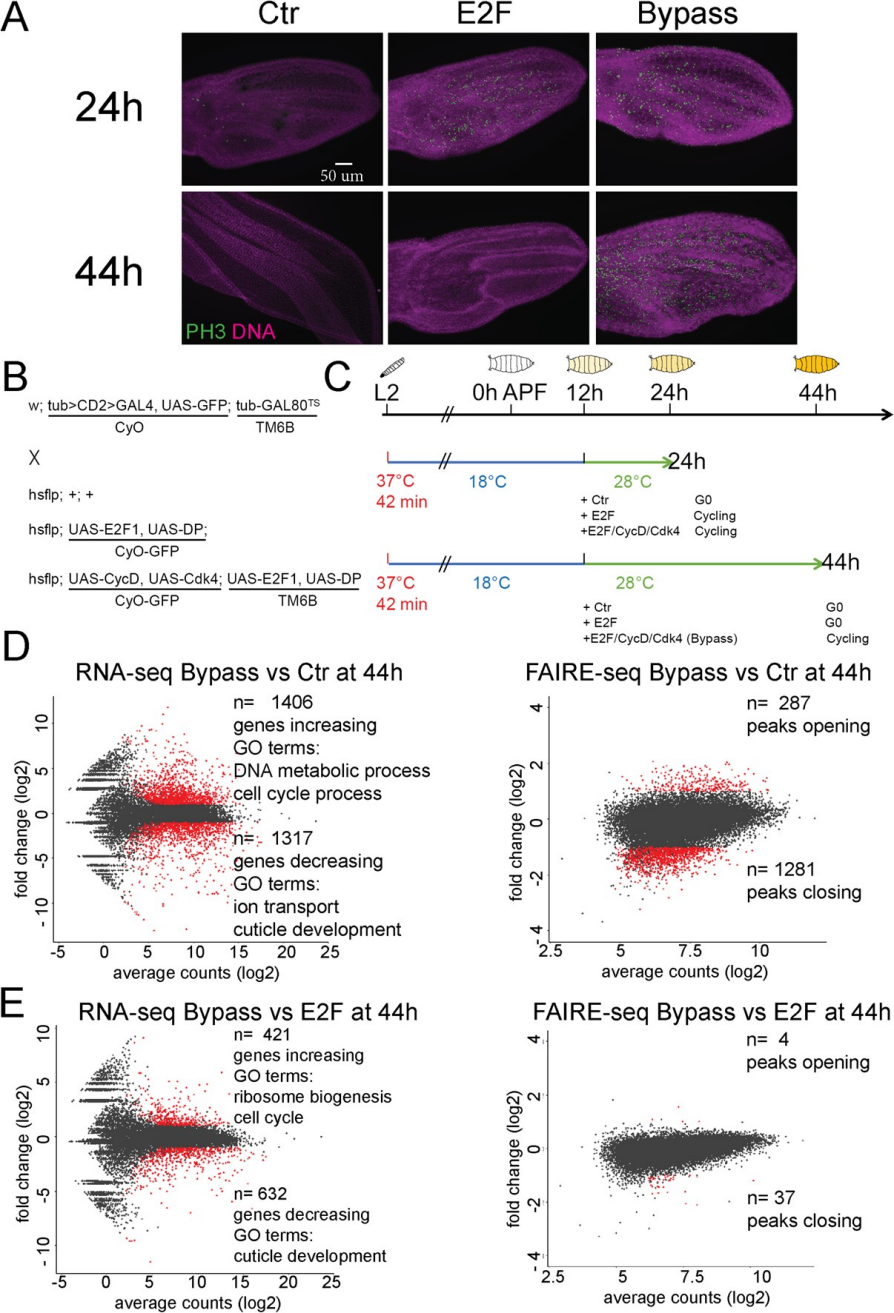
Global impacts of cell cycle exit disruption on gene expression and open chromatin. (A) G0 can be delayed to 36 h or bypassed beyond 50 h through short-term expression of E2F or E2F + CycD/Cdk4. Transgenes were overexpressed in the dorsal layer of wing epithelia under the control of Apterous-Gal4/Gal80^TS^ from 12 h APF. Twenty-four-hour and 44-h wings were immunostained for ph3. (B, C) Genotype and scheme of RNA-seq and FAIRE-seq experiments to disrupt cell cycle exit during metamorphosis. (D, E) MA plots of RNA and FAIRE changes comparing bypassed exit (E2F + CycD/cdk4) between Ctr (D) and delayed cell cycle exit (E2F) (E) at 44 h. Abundant changes in expression of cell cycle genes, ribosome biogenesis, and cuticle formation genes are observed, while chromatin accessibility is nearly identical between conditions in which cells enter a delayed G0 versus continue cycling. The underlying data in Fig 3D and 3E can be found within S3 Data. APF, after puparium formation; Cdk, cyclin-dependent kinase; Ctr, control; CycD, Cyclin D; CyO, Curly O balancer; DP, dimerization partner; E2F, E2F transcription factor; FAIRE, formaldehyde-assisted isolation of regulatory elements; FAIRE-seq, FAIRE sequencing; Gal80^TS^, temperature-sensitive Gal80; GO, Gene Ontology; hsflp, heatshock-flippase; MA plot, scatter plot onto M (log ratio) and A (mean average) scales; ph3, phosphohistone H3; RNA-seq, RNA sequencing; TM6B, TM6B balancer; UAS, upstream activation sequence.

We observed that chromatin dynamics at master regulator cell cycle genes is coordinated with cell cycle changes during metamorphosis. However, a key question is whether the closing of chromatin at these genes is a cause or consequence of cell cycle exit. To address this question, we took advantage of conditions in which cell cycle exit in the pupal wing can be either temporarily delayed or bypassed for a prolonged period without preventing metamorphosis or terminal differentiation. In brief, overexpression of the activator E2F complex during the final cell cycle delays cell cycle exit and causes an extra cell cycle during the 24–36 h window, whereas overexpression of E2F + CycD/Cdk4 during this same period causes multiple rounds of extra cell division and effectively bypasses cell cycle exit until well after 50 h [25]. We used the Gal4/UAS system in combination with a temperature-sensitive tub-Gal80 (Gal80^TS^) to limit the perturbation of the cell cycle from 12 to 24 or 44 h APF. This allows metamorphosis to initiate properly yet effectively delays G0 by one extra cell cycle or bypasses G0 with multiple rounds of extra division (Fig 3A, S6 Fig). We dissected 24 h or 44 h pupal wings under the delayed (E2F) or continued cycling (E2F + CycD) conditions and performed genome-wide RNA-seq and FAIRE-seq analysis (Fig 3B and 3C). Importantly, at 44 h when the E2F expressing wings are postmitotic, the E2F + CycD wings are still cycling, allowing us to distinguish the effects of E2F overexpression from those of preventing cell cycle exit.

E2F or E2F + CycD expression was sufficient to alter the expression of several hundred genes at 24 h and over 1,500 by 44 h (Fig 3D, S7 Fig). Despite the dramatic changes in gene expression, there were strikingly few changes in FAIRE peak accessibility, with only a handful of peaks increasing accessibility at the 24-h time point and up to 287 peaks increasing accessibility at the 44-h time point (Fig 3D, S7 Fig). Gene Ontology (GO) term enrichment analysis under both conditions revealed that the up-regulated genes are highly enriched for those associated with the cell cycle, whereas down-regulated genes are highly enriched for genes involved in cuticle development. To determine whether these few accessibility changes were due to the ectopic E2F activity or the continued ectopic proliferation itself, we compared chromatin accessibility changes between E2F and E2F + CycD wings at 44 h (Fig 3E). Whereas RNA-seq revealed differential effects on the expression levels of several hundred genes involved in the cell cycle, ribosome biogenesis, and cuticle development, FAIRE-seq revealed almost no changes in chromatin accessibility between these two conditions. This is remarkable considering that wings expressing E2F at 44 h are fully postmitotic, whereas wings expressing E2F + CycD continue to proliferate (S6 Fig). This demonstrates that the cycling status of differentiating cells has little direct effect on chromatin accessibility at potential regulatory elements.

**Fig 4:**
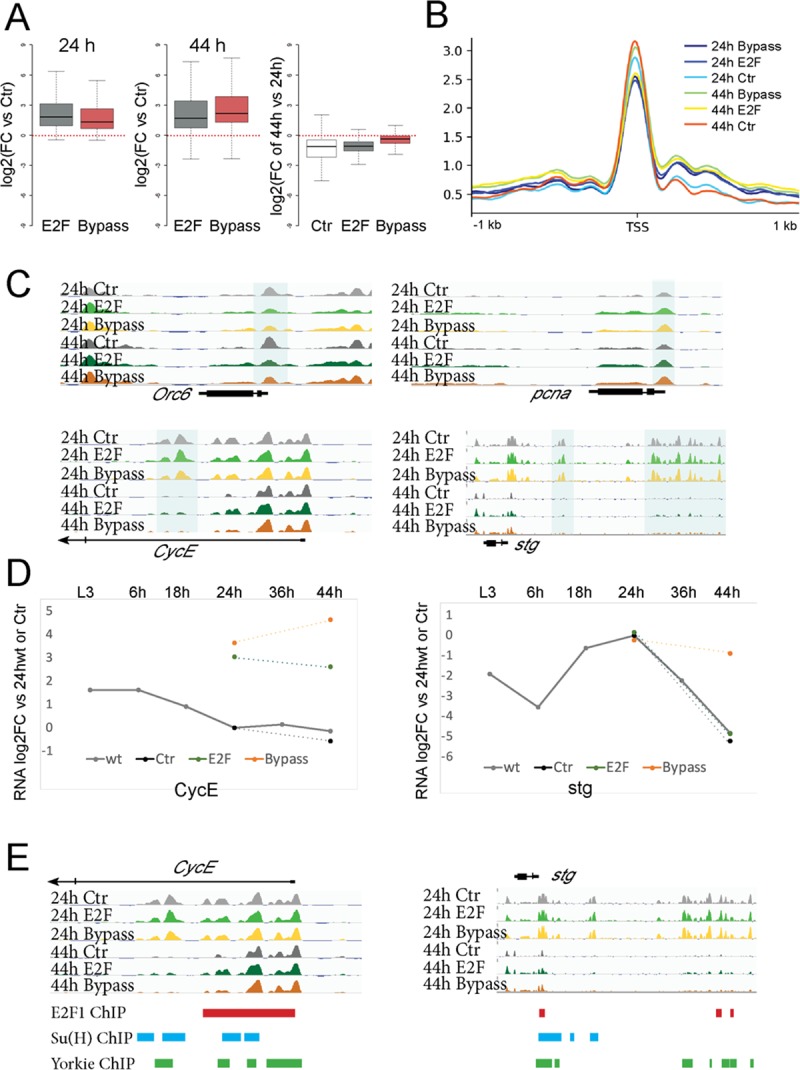
Distal enhancer accessibility of complex cell cycle genes is developmentally controlled and independent of cell cycling status. (A) Expression of cell cycle genes is increased when we delay or bypass cell cycle exit (log2 fold change for cell cycle genes versus ctrs expressing GFP). (B) Line plots of average FAIRE signal for cell cycle genes. Accessibility at most cell cycle genes’ TSS is slightly decreased when cell cycle exit is delayed (44 h E2F expression, *p*-value = 1.004 × 10^−5^, Mann-Whitney U Test). (C) Regulatory elements for simple cell cycle genes (*orc6*, *pcna*) remain accessible independent of cycling status. Complex cell cycle genes (*cycE*, *stg*) lose accessibility at regulatory regions independent of cycling status. (D) Expression of *cycE* and *stg* during metamorphosis (gray line, compared with 24-h wings) and genetic manipulations (colored dots, compared with 24-h ctr wings). *stg* possesses higher barrier for activation compared with *CycE*. Closed stg regulatory elements likely prevent *stg* expression in the late robust E2F-expressing wings, whereas E2F + CycD/Cdk4 can overcome this proliferation barrier. (E) Dynamic distal wing enhancers and TSS proximal regions for *cycE* and *stg* contain sites that bind E2F1, Su(H), and Yorkie based on ChIP in cycling tissues. The underlying data in Fig 4D can be found within S4 Data. Cdk, cyclin-dependent kinase; ctr, control; ChIP, chromatin immunoprecipitation; CycD, Cyclin D; CycE, Cyclin E; E2F, E2F transcription factor; FAIRE, formaldehyde-assisted isolation of regulatory elements; FC, fold change; GFP, green fluorescent protein; orc6, origin recognition complex subunit 6; pcna, proliferating cell nuclear antigen; stg, string; Su(H), Suppressor of Hairless; TSS, transcription start site; wt, wild type.

Despite the up-regulation of hundreds of cell cycle–related genes at both 24 and 44 h (Figs 3D, 3E and 4A), we observed little effect on their accessibility (Fig 4B). Examples of simple (*origin recognition complex subunit 6* [*orc6*] and *pcna)* and complex cell cycle genes (*cycE* and *stg)* showed minor changes in chromatin accessibility when cell cycle exit was delayed or disrupted. Importantly, the closing of *cycE* and *stg* distal enhancers proceeds with normal timing despite the delay or bypass of cell cycle exit (Fig 4C). This demonstrates that we have uncoupled differentiation from cell cycle exit and that the closing of regulatory elements at complex cell cycle genes is developmentally controlled and independent of cell cycling status. Importantly, the closing of distal regulatory elements seems to prevent the activation of *stg* by ectopic E2F but not E2F + CycD (Fig 4D). This suggests that the continued closing of regulatory elements at these cell cycle genes underlies the increased robustness of the G0 state at these later time points.

We next looked more closely at the 1,053 differentially regulated genes in E2F versus E2F + CycD conditions at 44 h to understand how these genes change expression with so few chromatin accessibility changes. We find that RNA levels for almost half of these (501 genes) are also altered in the E2F-only condition, but to a lesser degree than in the E2F + CycD condition. For these genes, we also see accessibility changes when comparing E2F + CycD to the wild-type control, but not when we compare E2F + CycD with E2F only (Fig 3E). This is because these genes show similar accessibility changes in the E2F-only condition, even though their RNA is more strongly altered in the E2F + CycD condition. This suggests that the dynamic range for the RNA signal is much greater than that for FAIRE signal, and these genes may be significantly differentially regulated at the RNA level under the E2F + CycD condition, even though their accessibility has not significantly changed from the E2F-only condition.

Similarly, an additional 119 genes in this group are bona fide RB/E2F or dREAM/ Myb-Muv B (MMB) target genes that are only mildly affected in the E2F-only condition and do not pass our RNA-seq cutoff [9,55,56]. We find that overexpression of E2F + CycD leads this category of genes to become more strongly and more rapidly induced than E2F alone, pushing them past our threshold for significance under the E2F + CycD condition versus the E2F-only condition. This category of genes may become strongly activated by the ectopic CycD/Cdk4 activity, facilitating the switch between repressive E2F complexes and E2F activator occupancy at the same binding sites [57], and therefore, no changes in accessibility need to occur for activation of expression. We also find 42 genes involved in ribonucleoprotein complexes or ribosome biogenesis to be altered by E2F + CycD, some of which have been shown to be repressed directly by Rbf2 in an E2F-independent manner [56]. The addition of CycD/Cdk4 may lead to derepression of these genes through phosphorylating Rbf2 and disrupting its chromatin binding, although at this time, the nature of these E2F-indpendent complexes and whether they alter chromatin accessibility is unknown.

The remaining 354 genes affected specifically when cell cycle exit is bypassed are enriched for GO terms involved in RNA metabolism (*p* < 9.81^−6^) and noncoding RNA (ncRNA) processing (*p* < 4.76^−5^), and most of these genes (199) are down-regulated. We do not clearly understand how these genes are regulated by the cell cycle at this time. We speculate that, for these genes, adding CycD/Cdk4 activity could have direct or indirect effects on additional transcriptional regulators that do not alter chromatin accessibility, possibly by acting through or near promoter proximal sites, or CycD/Cdk4 activity could have effects on transcript stability. Consistent with these ideas, this group of genes includes 35 transcription or RNA polymerase II binding factors (e.g., DRE-binding factor [*Dref*], Mediator complex components, *retained*, *deadpan*, *extra-extra*, *teashirt*, *Activating transcription factor 3*, *salivary gland-expressed bHLH*, *Nuclear factor I*) and 17 regulators of RNA splicing and stability (such as *partner of drosha*, Piwi-interacting RNA pathway components, *lariat debranching enzyme*, *Small ribonucleoprotein particle protein SmB*, *bruno 1*). We also cannot rule out the possibility that significant changes in RNA-seq signal may result from a small subset of cells in the wing that change gene expression and accessibility but that cannot be captured by the limited dynamic range of the chromatin accessibility signal from the whole tissue.

### Delaying cell cycle exit impacts a subset of genes involved in wing terminal differentiation

In contrast to the minimal effects on cell cycle genes, the largest impact of delaying or disrupting cell cycle exit on chromatin was the loss of accessibility at over 1,000 genomic sites at 44 h (S7 Fig). This could be caused by either chromatin remodeling to close accessible sites at 44 h or a failure to open sites that should become accessible. To address which of these scenarios is correct, we examined the dynamics of peaks influenced by E2F or E2F + CycD during the wild-type time course (S8 Fig). Notably, peaks that are less accessible in E2F-expressing wings are closed at 36 h but highly accessible at 44 h in wild-type wings. This suggests that delaying or disrupting cell cycle exit results in a failure to open a specific subset of regulatory elements between 36 h and 44 h. Our data suggest that this failure to open specific elements is due to the ectopic E2F activity rather than ectopic proliferation itself, as there are strikingly few chromatin accessibility changes between E2F and E2F + CycD wings at 44 h (Fig 3E).

**Fig 5:**
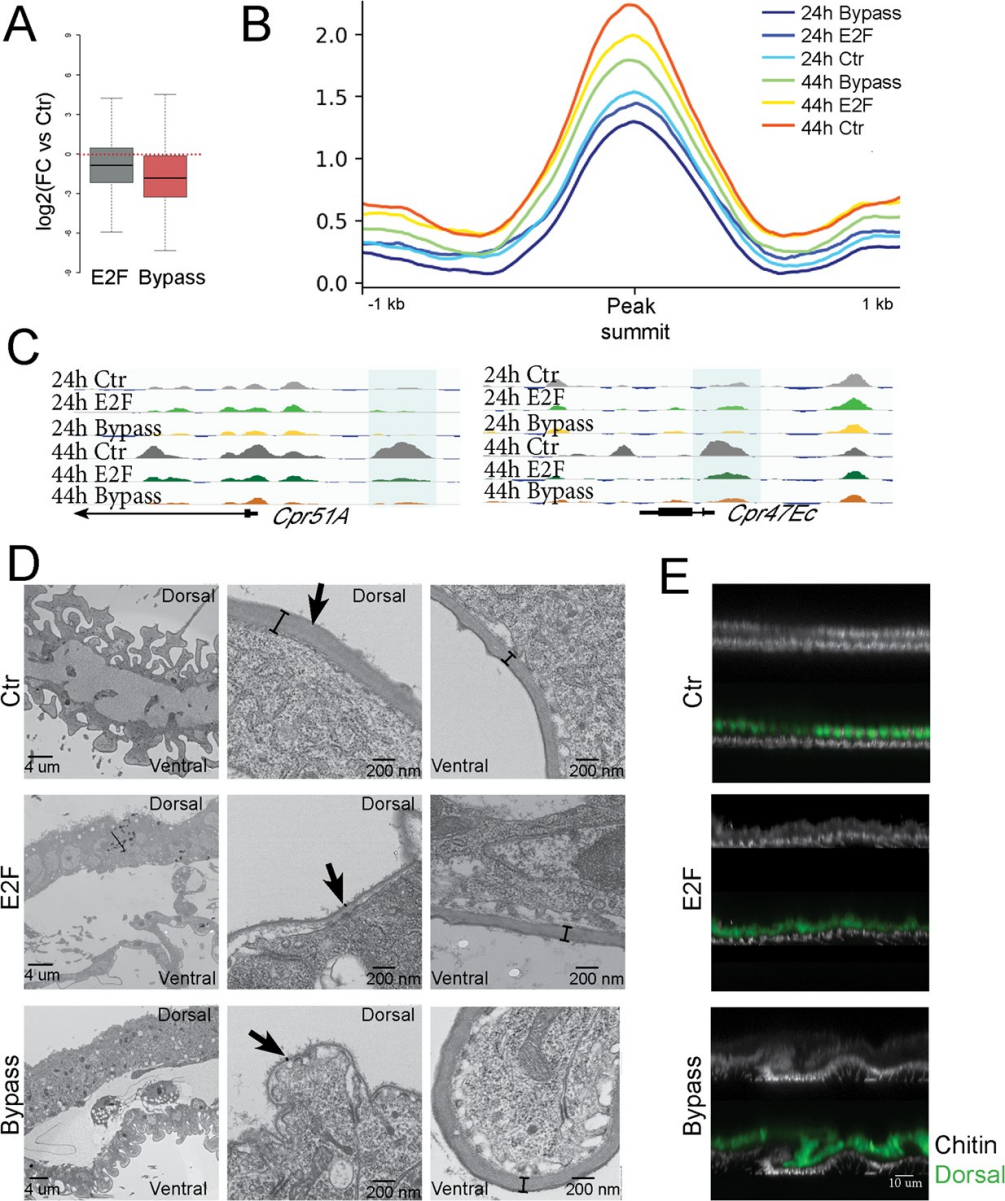
Compromising cell cycle exit impacts chromatin accessibility and gene expression at a subset of wing terminal differentiation genes. (A) log2 fold changes in RNA and (B) line plots of average FAIRE signal for genes involved in cuticle formation and differentiation. Preventing cell cycle exit reduces their expression and chromatin accessibility (E2F + CycD/Cdk4 at 44 h, *p*-value = 0.0046, Mann-Whitney U Test). (C) Selected cuticle protein genes exhibiting a failure to open potential regulatory elements at 44 h when cell cycle exit is delayed or bypassed. (D, E) Representative TEM (D) and chitin staining (E) of 64-h wings that delayed or bypass cell cycle exit in the dorsal wing epithelium using *Apterous*-Gal4/Gal80^TS^ to activate E2F or E2F + CycD/Cdk4 expression during the final cell cycle. Extracellular matrix formation and chitin deposition are disrupted when cell cycle exit is compromised. The underlying data in Fig 5A can be found within S5 Data. Cdk, cyclin-dependent kinase; Cpr, Cuticular protein; Ctr, control; CycD, Cyclin D; E2F, E2F transcription factor; FAIRE, formaldehyde-assisted isolation of regulatory elements; FC, fold change; Gal80^TS^, temperature-sensitive Gal80; TEM, transmission electron microscopy.

The loci that fail to open when cell cycle exit is disrupted are located near genes enriched for roles in cuticle formation and deposition and wing terminal differentiation. Consistent with this, expression levels of genes involved in wing cuticle formation are reduced when cell cycle exit is delayed or disrupted (Fig 5A), and chromatin accessibility at their potential regulatory elements is reduced (Fig 5B and 5C). Together, our results indicate that delayed cell cycle exit and ectopic E2F activity compromise the opening and activation of a portion of the wing terminal differentiation program.

To determine whether ectopic E2F activity impacts wing cuticle formation, we expressed E2F or E2F + CycD in the dorsal layer of the wing epithelium beginning at 12 h APF using *Apterous*-Gal4/Gal80^TS^. We examined the cuticle formation at 64 h by transmission electron microscopy (TEM) (Fig 5D). Pupal wings are normally composed of two thin monolayers of epithelial cells, and expression of E2F or E2F + CycD led to an obvious thickening of the epithelium due to extra cell divisions in the dorsal side. The cuticle layer on the dorsal side of the wing was much thinner than normal, and the effect on the cuticle was compartment autonomous, leaving ventral wing cuticle unaffected. We next examined the deposition of chitin, the key component of insect cuticle, through calcofluor staining (Fig 5E). Chitin staining in the dorsal wing, where E2F or E2F + CycD was expressed, was much weaker than in the ventral. Thus, ectopic E2F activity delays and disrupts the adult wing cuticle program in a compartment-autonomous manner.

### Disrupting cell cycle exit alters chromatin dynamics at specific ecdysone target genes

**Fig 6:**
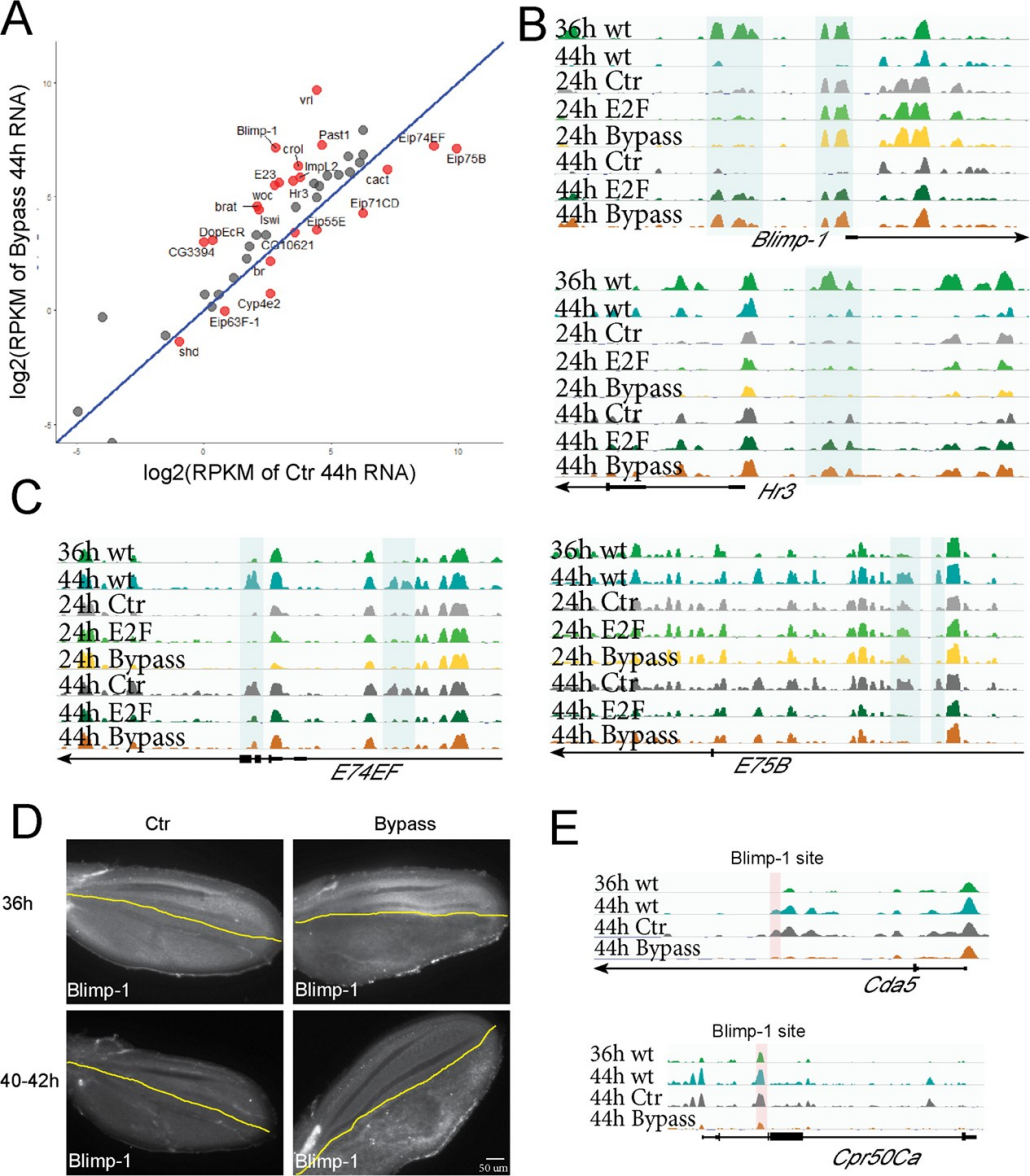
Bypassing cell cycle exit disrupts chromatin dynamics at ecdysone target genes and alters their expression. (A) Scatterplot of ecdysone-responsive genes in 44-h wings under conditions that bypass cell cycle exit versus normal exit. Genes with significant changes in expression are labeled in red. (B, C) Chromatin regions of *Blimp-1*, *Hr3*, *E74*, and *E75* fail to close or open at 44 h when cell cycle exit is compromised. (D) Blimp-1 antibody staining of wings at 36 h and 40–42-h wings with normal cell cycle exit (Ctr) or bypassed cell cycle exit in the posterior (using *engrailed*-Gal4/Gal80^TS^). Compromising cell cycle exit delays the activation of Blimp-1 in a compartment-autonomous manner. (E) Peaks that fail to open at 44 h from cuticle-development genes harbor high-scoring Blimp-1 binding sites. The underlying data in Fig 6A can be found within S6 Data. Cda5, Chitin deacetylase-like 5; Cpr, Cuticular protein; Ctr, control; E2F, E2F transcription factor; Hr3, Hormone receptor 3; Gal80^TS^, temperature-sensitive Gal80; RPKM, reads per kilobase of transcript, per million mapped reads; TEM, transmission electron microscopy; wt, wild type.

Our findings suggest the existence of cross talk between cell cycle exit and the later terminal differentiation gene expression programs. We next sought to identify the factors mediating this cross talk. We noticed that several ecdysone target genes were amongst the genes impacted by the delay or disruption of cell cycle exit (S8 Fig). Ecdysone signaling coordinates developmental timing between tissues during metamorphosis. We systematically examined ecdysone target genes and found that genes such as *Blimp-1*, *Hormone receptor 3* (*Hr3*), and *crooked legs* (*crol*) were expressed at significantly higher levels at 44 h when cell cycle exit was disrupted, whereas the expression of *E74EF*, *E75B*, and *E71CD* was reduced (Fig 6A). During the normal time course, *Blimp-1*, *Hr3*, and *crol* exhibit peak expression at 36 h and plummet by 44 h, whereas *E74EF*, *E75B*, and *E71CD* normally peak at 44 h. Thus, the disruption of cell cycle exit leads to a delay in the shutoff of *Blimp-1*, *Hr3*, and *crol* and delayed up-regulation of *E74EF*, *E75B*, and *E71CD*. When we investigated chromatin accessibility at these genes, we found that specific potential regulatory elements for Blimp-1 and Hr3 failed to close at 44 h when cell cycle exit was disrupted, whereas specific potential regulatory elements at E75B and E74EF failed to open (Fig 6B and 6C). Our results suggest a model in which ectopic E2F activity leads to delays in chromatin remodeling at specific ecdysone target genes, delaying their proper expression dynamics.

We reasoned that these alterations in transcriptional regulators downstream of ecdysone signaling could lead to the alterations in chromatin accessibility for wing terminal differentiation genes when cell cycle exit is disrupted. Consistent with this, the Blimp-1 binding motif is significantly enriched in FAIRE peaks that are differentially regulated under conditions that delay or disrupt cell cycle exit (S9 Fig). Several genes important for cuticle development such as *Chitin deacetylase-like 5* (*Cda5*), *Cpr50Ca*, *Cpr47Ec*, and *TweedleT* (*TwdlT*) harbor high-scoring Blimp-1 binding sites and are likely direct Blimp-1 targets (S9 Fig). Their peaks exhibit temporal dynamics consistent with a model in which Blimp-1 either binds closed chromatin at 36 h and facilitates subsequent chromatin opening at 44 h or in which high Blimp-1 binding at 36 h somehow maintains closing that is lost when Blimp-1 levels plummet at 44 h (Fig 6E). The temporal and spatial resolution of our FAIRE time course is not sufficient to distinguish between these two scenarios. Interestingly, we also found a high-scoring *Blimp-1* site in E74EF, suggesting its temporal regulation is also dependent on Blimp-1.

**Fig 7:**
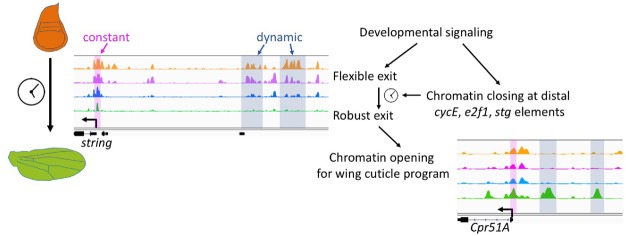
A model for the developmental coordination of cell cycle exit and chromatin accessibility. Potential regulatory elements at complex cell cycle genes such as *stg* become inaccessible in a developmentally controlled manner during robust G0. This may limit their activation in response to proliferative signals. Delaying or disrupting cell cycle exit impacts the subsequent opening of chromatin at genes in the wing terminal differentiation program that are potentially controlled via transcription factors downstream of ecdysone signaling. Cpr, Cuticular protein; cycE, cyclin E; E2F, E2F transcription factor; stg, string.

We considered the possibility that our genetic disruption of cell cycle exit could have nonautonomous effects that impact the timing or production of the ecdysone signal itself, leading to alterations in chromatin remodeling at specific targets. We therefore tested whether our manipulations of cell cycle exit impact Blimp-1 expression nonautonomously. For this, we expressed E2F + CycD specifically in the posterior compartment of the pupa wing using the *Engrailed*-Gal4/Gal80^TS^ system. Under these conditions, only the posterior wing continues to proliferate, whereas the anterior wing becomes postmitotic with the normal timing [25]. We found that when we disrupted cell cycle exit in the posterior wing only, Blimp-1 protein levels were reduced at 36 h but higher at 40–42 h, consistent with the delay in *Blimp-1* activation we observed by RNA-seq. Importantly, Blimp-1 levels were unaffected in the anterior wing, showing the normal increase in Blimp-1 levels at 36 h and drop in levels at 44 h. This demonstrates that disrupting cell cycle exit impacts the timing of ecdysone target gene expression in a compartment-autonomous manner (Fig 6D), consistent with our findings on compartment-specific effects on cuticle formation (Fig 5E). Our data are consistent with a model in which the *cis*-regulatory DNA at genes encoding hormone-regulated transcription factors such as Blimp-1 responds to ectopic cell cycles or E2F activity to coordinate cell cycle exit with later steps of terminal differentiation downstream of the hormone pulses. This in turn leads to delays in chromatin remodeling at their targets and altered expression dynamics of downstream wing terminal differentiation genes (Fig 7).

## Discussion

### Most chromatin and gene expression changes in the wing during metamorphosis are independent of cell cycling status

A striking feature of the *Drosophila* wing during metamorphosis is the coordination between the cell cycle and tissue morphogenetic changes. We hypothesized that the synchronous exit from the cell cycle may impact chromatin accessibility and lead to widespread gene expression changes to coordinate differentiation with cell cycle exit. However, this is not the case. When we compromise cell cycle exit, we observe relatively few changes in chromatin accessibility. We therefore conclude that the majority of chromatin accessibility dynamics during metamorphosis are developmentally programmed and occur coincident with cell cycle exit but are independent of the transition from a proliferating to a postmitotic state.

### Chromatin accessibility changes at a subset of cell cycle genes during metamorphosis

Terminal differentiation and the transition from proliferation to a postmitotic state are usually coupled during development. Cell cycle arrest has been proposed to be essential for terminal differentiation in several cell types by promoting or maintaining the proper expression of late differentiation genes [58–61]. Although studies in other contexts have shown that cell cycle exit and overt terminal differentiation can be separable [15,62–65], in this study, we have comprehensively characterized the gene expression and gene regulatory mechanisms underlying these two processes by examining the transcriptome and open chromatin landscape changes during cell cycle exit. Our study reveals that during wing differentiation, chromatin accessibility and gene expression changes are temporally coordinated and that distal regulatory elements at a subset of critical cell cycle genes (*cycE*, *stg*, *e2f1*) become inaccessible during terminal differentiation, even when cell cycle exit is compromised (Figs 3,4).

We suggest that these changes in chromatin accessibility at essential and rate-limiting cell cycle genes provide additional barriers to cycling and provide a molecular explanation for the robust G0 state we observe after 36 h APF. Notably, the closed distal regulatory elements at *cycE*, *stg*, and *e2f1* in robust G0 contain sites known to bind Suppressor of Hairless (Su[H]), E2F, and Yorkie in cycling tissues (Fig 4E) [66–68], in addition to predicted sites for transcription factors of pathways that promote proliferation in undifferentiated wings. Their closing likely explains why pupal wings after 36 h fail to proliferate in response to many hyperplastic signals, including direct activation of Notch or Yorkie [69].

Although the distal pupal wing enhancers for *cycE* and *stg* remain inaccessible when we disrupt cell cycle exit (Fig 4), ectopic E2F + CycD can reactivate *cycE* and *stg* expression at 44 h to support continued cycling (Fig 4D). This is consistent with our previous finding that ectopic CycE + Stg is required in addition to E2F to keep cells in the wing cycling past robust G0 or to induce cell cycle reentry from an established postmitotic G0. We suggest that ectopic E2F + CycD acts through accessible TSS proximal regulatory sites to “short-circuit” robust G0 and reactivate *cycE* and *stg* expression. Further work on these potential regulatory elements will be required to examine why TSS proximal binding allows for activation of *cycE* and *stg* by E2F + CycD but not other regulators.

The addition of ectopic CycD is essential for the reactivation of *stg* expression at 44 h and continued cycling. Ectopic E2F alone neither reactivates *stg* nor supports continued cycling, despite increasing the expression of many other cell cycle genes (Fig 4). Why is adding CycD required for reactivation of *stg* expression? The most common model for CycD function is that it phosphorylates Rbf and weakens RB-mediated repression of E2F, thereby compromising DREAM repressive function. This would suggest that ectopic CycD may be needed to overcome additional dREAM repressive barriers at specific targets. However, recent work has suggested CycD activity may also play other roles to promote cell cycle entry from G0 [70]. Further work to examine how *cycE* and *stg* are activated in this context and the proteins that facilitate this during metamorphosis will be necessary.

Our model is consistent with recent findings in *C*. *elegans* and other contexts that chromatin remodeling plays an important role in ensuring cell cycle exit during differentiation [19,71]. However, by specifically disrupting cell cycle exit rather than chromatin remodeling, as in previous studies, we are able to largely uncouple terminal differentiation and cell cycle arrest in the fly wing. This allowed us to distinguish changes in chromatin accessibility at a subset of late differentiation genes that are dependent upon proper cell cycle arrest from those throughout the majority of the genome, including cell cycle genes that are independent from arrest.

Although we do not know the chromatin remodelers responsible for the closing of the distal regulatory elements at *cycE*, *stg*, and *e2f1*, we do find conserved Ecdysone receptor (EcR) binding sites that exhibit peaks of EcR binding during pupal stages according to Model Organism Encyclopedia Of DNA Elements (ModENCODE) data [72]. An attractive model is that the strong peak of ecdysone occurring at 24 h triggers EcR complexes to recruit chromatin remodelers to subsequently modulate elements at differentiation genes and complex cell cycle genes to coordinate differentiation with cell cycle exit. As our disruptions of cell cycle exit do not seem to impact the pulse of ecdysone at 24 h, closing of chromatin via this mechanism would proceed independent of cell cycling status and act to limit cell cycle entry even in the presence of strong ectopic cell cycle activation.

### Preventing cell cycle exit compromises a portion of the wing terminal differentiation program

Our results reveal that most chromatin accessibility changes at potential regulatory elements in fly wings are developmentally regulated and change independent of cell cycling status. However, compromising cell cycle exit does alter chromatin accessibility at a small subset of temporally regulated transcription factors that impact the proper timing of the wing cuticle differentiation program (Fig 6). We find that compromising cell cycle exit somehow cell autonomously delays the temporal gene expression and chromatin remodeling cascade downstream of ecdysone signaling in the wing (Fig 7). Our work implies an important role for Blimp-1 in the wing cuticle differentiation program. Blimp-1 is directly induced by ecdysone and is a transcriptional repressor that has been well studied for silencing *ftz transcription factor 1* (*ftz-f1*) at the onset of metamorphosis, as well as regulating cuticle formation in the fly embryo [73–75]. Here, we show that *Blimp-1* is also highly expressed at 36-h-APF wings, following the second and highest pulse of ecdysone during metamorphosis. *Blimp-1* is then immediately silenced by 44 h. We found that dynamic chromatin regions that open at 44 h are enriched for Blimp-1 binding sites. Some of these sites are potential regulatory elements for cuticle genes and other ecdysone targets such as *E74EF* (S9 Fig). Because these regions are closed when Blimp-1 is present and only open after Blimp-1 goes away, we propose that Blimp-1 blocks the accessibility of these dynamic regulatory elements. Consistent with this model, we also identified Blimp-1 binding sites at a dynamic open region at the *ftz-f1* locus. This region is transiently open at 6 h when Blimp-1 is absent and *ftz-f1* is expressed, mirroring the pattern of accessibility for the potential Blimp-1 site at *E74EF* at 44 h. Future work will focus on revealing the proximal factor that acts on chromatin accessibility at Blimp-1 regulatory elements to link cell cycling status with the temporal wing differentiation program.

## Materials and methods

### Fly stocks and genetics

FAIRE and RNA-seq samples with genetic manipulations are as follows:

*w/ y*, *w, hs-FLP; tub>CD2>GAL4, UAS-GFP/ +; tub-gal80TS/ +*

*w/ y*, *w, hs-FLP; tub>CD2>GAL4, UAS-GFP/ UAS-E2F1, UAS-DP; tub-gal80TS/ +*

*w/ y*, *w, hs-FLP; tub>CD2>GAL4, UAS-GFP/ UAS-CycD, UAS-Cdk4; tub-gal80TS/ UAS-E2F1, UAS-DP*

Transgenes are described in [69]. The *tub>CD2>Gal4* is from [76], and UAS E2F1, DP, CycD, and Cdk4 are from [77].

Crosses were set up at 25°C. Second instar larva (L2) were heat shocked at 37°C for 42 min and then kept at 18°C. White prepupae were collected for staging and kept at 18°C until the equivalent of 12 h APF at 25°C. Then, pupae were shifted to 28°C until the equivalent of 24 h APF or 44 h APF at 25°C for dissection. Pupae develop 2.2 times more slowly at 18°C than at 25°C and 1.2 times faster at 28°C than at 25°C. All time points were adjusted to equivalent stages at 25°C for figures.

Genotypes for TEM, chitin, and phosphohistone H3 (PH3) staining are as follows:

*w/y*, *w, hs-FLP; Ap-GAL4, UAS-GFP/ +; tub-gal80TS/ +*

*w/y*, *w, hs-FLP; Ap-GAL4, UAS-GFP/ UAS-E2F1, UAS-DP; tub-gal80TS/ +*

*w/y*, *w, hs-FLP; Ap-GAL4, UAS-GFP/ UAS-CycD, UAS-Cdk4; tub-gal80TS/ UAS-E2F1, UAS-DP*

Crosses were set up and kept at 18°C. White prepupae were collected and aged to the equivalent of 12 h APF and then shifted to 28°C until the equivalent of 24 h APF, 44 h APF (for PH3 staining), or 64 h APF (for TEM and chitin staining).

Genotypes for Blimp-1 antibody staining are as follows:

*w/y*, *w, hs-FLP; en-GAL4, UAS-GFP/ +; tub-gal80TS/ +*

*w/y*, *w, hs-FLP; en-GAL4, UAS-GFP/ UAS-CycD, UAS-Cdk4; tub-gal80TS/ UAS-E2F1, UAS-DP*

*w/y*, *w, hs-FLP; en-GAL4, UAS-GFP/ Blimp-1^RNAi^ (BL 57479), UAS-DP; tub-gal80TS/ +*

*w/y*, *w, hs-FLP; en-GAL4, UAS-GFP/ +; tub-gal80TS/ white^RNAi^*

Crosses were set up and kept at 18°C. White prepupae were collected and shifted to 28°C until the equivalent of 36 h APF or 40–42 h APF for immunostaining.

Genotypes for PCNA reporter assay are as follows:

*PCNA-EmGFP/ y*, *w, hs-FLP; +; act>CD2>gal4, UAS-RFP/+*

*PCNA-EmGFP/ y*, *w, hs-FLP; UAS-E2F1, UAS-DP /+; act>CD2>gal4, UAS-RFP/+*

*PCNA-EmGFP/ y*, *w, hs-FLP; +/UAS-CycD, UAS-Cdk4; act>CD2>gal4, UAS-RFP/ UAS-E2F1, UAS-DP*

The *PCNA-EmGFP* line is described in [78]. Crosses were set up and kept at 25°C. White prepupae were collected and incubated to 26 h APF and then heat shocked at 37°C for 12 min and incubated at 25°C until 42 h APF for dissection.

### Enhancer-Gal4 reporters

Transgenic flies were crossed with UAS-GFP (*cpr51A* region, VT016704) or UAS-destabilized GFP (*stg* region, BL45586, and *e2f1* region, VT045332) and incubated at 25°C. Then larval or pupal samples (staged from white prepupae) were dissected and immunostained for GFP. The cycE-GFP transgenic reporter was kindly provided by Dr. Sarah Bray [66]. Images were taken with the same intensity and gain across time points for comparison.

### Sample preparation and data analysis for high-throughput sequencing

FAIRE samples and RNA samples were prepared as described previously [27]. FAIRE-seq reads were aligned to the dm6 reference genome using Bowtie2 [79]. FAIRE-seq peak calling was performed using MACS2 and PePr [80,81] with q-value threshold at 0.01, and only common peaks from both programs were utilized for further analysis. Z-scores were calculated using the mean and standard deviation per chromosome arm. High-fidelity peaks were chosen from peaks with maximal Z-score larger than 2. FAIRE-seq line plots were generated using deepTools [82]. FAIRE-seq peaks were visualized using Integrative Genomics Viewer [83]. DNA-binding motifs used for enrichment analysis were obtained from FlyFactorSurvey [84]. Motif de novo discovery, comparison with known motif, and motif enrichment analysis were done using the MEME tool, TOMTOM tool, and AME tool in MEME suite [85]. Annotation of FAIRE peaks were carried out by assigning peaks to nearest TSS in R package ChIPpeakAnno [86]. RNA-seq reads were aligned to the dm6 reference genome using STAR and further counted using HTSeq [87,88]. RPKM values of RNA-seq were calculated through Cufflinks [89]. Differentially expressed genes were defined as those having RPKM > 1 in at least one stage and changing by at least 2-fold between pairwise time points. GO analysis was performed using Database for Annotation, Visualization, and Integrated Discovery (DAVID) [90]. All the statistical comparisons are carried out in DEseq2 [91].

### Immunostaining and microscopy

Immunostaining procedures were carried out as previously described [25]. Primary antibodies used in this study include the following: anti-phospho-Ser10 histone H3, 1:2,000 rabbit (Millipore #06–570) or mouse (Cell Signaling #9706); anti-GFP, 1:1,000 chicken (Life Technologies A10262) or 1:1,000 rabbit (Life Technologies A11122); and anti-Blimp-1, 1:500 rabbit (Active motif 61054). DNA was labeled by 1 μg/ml DAPI in 1× PBS, 0.1% Triton X for 10 min, and chitin was stained by 50 μg/ml Fluorescent Brightener 28 (Sigma-Aldrich, F3543) in 1× PBS, 0.1% Triton X for 10 min. Images were obtained using a Leica SP5 confocal (chitin staining) or Leica DMI6000B epifluorescence system. For comparisons of reporter expression at different time points, we used the same exposure time and gain/laser intensity. The images we show are representative images of expression patterns observed in all the wings, in which *n* = at least 2–5 for each stage. For the Blimp-1 staining, we performed an RNAi knockdown to verify the staining was Blimp-1 (S10 Fig), and all quantified images include the unaffected compartment as an internal control. For the Blimp-1 staining, we observed the phenotype shown 100% of the time, in which *n* = at least three wings for each stage. For chitin staining, we included an unaffected compartment as a stage-matched internal control and observed the phenotype shown 100% of the time (*n* = at least three wings for each stage). Quantification for all fluorescence images is presented in S11 Fig. Details for the intensity measurements are explained in the figure legend.

### TEM

Tissue was incubated in Karnovsky’s fixative for at least 1 h at room temperature and then overnight at 4°C. Samples were washed with 20× volume Sorenson’s buffer 3× before postfixing in 2% osmium tetroxide in Sorenson’s buffer for 1 h at room temperature. Tissue was again washed 3× with 20× volume Sorenson’s buffer and then dehydrated through ascending concentrations of acetone and embedded in EMbed 812 epoxy resin. Semithin sections were stained with toluidine blue for tissue identification. Selected regions of interest were ultra-thin sectioned to 70 nm in thickness and poststained with uranyl acetate and Reynolds lead citrate. They were examined using a JEOL JEM-1400 Plus TEM at 80 kV. TEM images showing the phenotype are representative images. We embedded three wings for sectioning and observed the phenotype shown in all wings.

## Supporting information

S1 Fig Gene expression is dynamic during metamorphosis. (A) The heatmap shows RNA log2 fold change (compared with L3) for the indicated stages. The pattern of RNA changes during metamorphosis is separated into 18 k-means clusters. (B) Line plots of the log2 fold change versus L3 for the indicated RNA clusters. Each gene is represented by a single gray line, and the average of all genes for the given cluster is plotted in red line. GO term enrichments are also shown along with their adjusted *p*-values. During metamorphosis, differentiation-related genes such as cuticle development are activated, whereas cell cycle genes are repressed. The underlying data for this figure can be found within S7 Data. GO, Gene Ontology.
(TIF)

S2 Fig Locations of dynamic versus static open chromatin. (A) Pie charts of the proportion of dynamic peaks and static peaks for each stage examined. Peaks without significant changes (<2-fold) between neighboring time points were defined as “static.” Peaks bearing changes >2-fold were defined as “dynamic.” (B, C) Radar charts display the distribution of indicated dynamic (B) and static (C) peak categories in different distances to TSS. (D, E) Radar charts display the distribution of indicated dynamic (D) and static (E) peak categories in cds, intron, nc genes, proximal promoter (−500 bp to 150 bp of TSS), UTRs, and intergenic regions. For dynamic peaks, “closing” is defined as peaks that decrease in accessibility by >2-fold compared with the previous stage; conversely, “opening” indicates peaks that increase in accessibility by >2-fold compared with the previous stage. The underlying data for this figure can be found within S7 Data. cds, coding sequence; nc gene, noncoding gene; TSS, transcription start site.
(TIF)

S3 Fig The majority of dynamic open chromatin is associated with gene activation rather than gene repression. Scatterplots of FAIRE peaks and corresponding genes with significant changes between two sequential stages. Significance is defined by 2-fold changes and adjusted *p*-values less than 0.05. FAIRE, formaldehyde-assisted isolation of regulatory elements.
(TIF)

S4 Fig Coordination of RNA and FAIRE peak changes grouped by RNA clustering. Trajectories of average changes between genes and their corresponding FAIRE peaks over the six stages for each of the 18 RNA clusters. Boxplot of the Pearson correlation coefficients between RNA and FAIRE for each RNA cluster is shown. The underlying data for this figure can be found within S7 Data. FAIRE, formaldehyde-assisted isolation of regulatory elements.
(TIF)

S5 Fig Coordination of RNA and FAIRE peak changes grouped by FAIRE peak clustering. Trajectories of average changes between FAIRE peaks and their corresponding genes over the six stages for each of the 18 FAIRE clusters. Boxplot of the Pearson correlation coefficients between RNA and FAIRE for each FAIRE cluster is shown. The underlying data for this figure can be found within S7 Data. FAIRE, formaldehyde-assisted isolation of regulatory elements.
(TIF)

S6 Fig Two stages of G0 in differentiating wings. (A) E2F or E2F/CycD/Cdk4 (bypass) was overexpressed in the dorsal layer of wing epithelia under the control of *Apterous*-Gal4/Gal80^ts^ from 12 h APF. The 24-h and 44-h wings were immunostained against PH3. (B) The number of PH3 spots of each wing is counted, and five wings for each genotype are quantified. (C) Cell cycle profile of the FAIRE samples that bypassed robust G0 by E2F/CycD/Cdk4 was examined by FACS. *p*-Values were determined by an unpaired *t* test; ****< 0.0001, ***< 0.001. The underlying data for this figure can be found within S7 Data. APF, after puparium formation; Cdk, cyclin-dependent kinase; CycD, Cyclin D; E2F, E2F transcription factor; FACS, Fluorescence-activated cell sorting; FAIRE, formaldehyde-assisted isolation of regulatory elements; Gal80^TS^, temperature-sensitive Gal80; PH3, phosphohistone H3.
(TIF)

S7 Fig RNA-seq and FAIRE-seq changes when G0 is delayed (E2F expression wings) or bypassed (E2F/CycD/Cdk4 expression wings). MA plots of RNA (A) and FAIRE (B) changes of 24- and 44-h wings compared with control. Genes and peaks that are significant in changes with 2-fold difference and adjusted *p*-value less than 0.05 are labeled in red. Cdk, cyclin-dependent kinase; CycD, Cyclin D; E2F, E2F transcription factor; FAIRE, formaldehyde-assisted isolation of regulatory elements; FAIRE-seq, FAIRE sequencing; MA plot, scatter plot onto M (log ratio) and A (mean average) scales; RNA-seq, RNA sequencing.
(TIF)

S8 Fig Bypassing cell cycle exit disrupts the temporal dynamics of chromatin accessibility at a subset of genes. (A, B) The heatmap shows the temporal dynamics during normal development for the peaks that are more accessible or less accessible at 44-h wings expressing E2F/CycD/Cdk4, plotted as a fraction of the maximum FAIRE rpkm value. Compromising G0 leads to the failure of proper closing of 36-h peaks as well as delayed opening of 44-h peaks. (C) Overlap between peaks that normally open at 36 h in wild-type and peaks more accessible at 44 h bypassed wings. (D) Overlap between peaks that normally open at 44 h in wild-type and peaks less accessible at 44 h bypassed wings. The underlying data for this figure can be found within S7 Data. Cdk, cyclin-dependent kinase; CycD, Cyclin D; E2F, E2F transcription factor; FAIRE, formaldehyde-assisted isolation of regulatory elements; rpkm, reads per kilobase of transcript, per million mapped reads.
(TIF)

S9 Fig Compromising G0 disrupts the temporal dynamics of potential Blimp-1 targets. (A) The Blimp-1 motif is enriched in the dynamic peaks disrupted by E2F or bypass found by AME analysis. (B) A list of genes containing peaks that fail to open at 44 h with high-scoring Blimp-1 binding sites. (C) Chromatin accessibility changes at *E74EF* and *ftz-f1* loci with Blimp-1 binding sites are shown. (D) Expression changes of *Blimp-1*, *ftz-f1*, and *E74EF* during normal development. The underlying data for this figure can be found within S7 Data. AME, Analysis of Motif Enrichment; E2F, E2F transcription factor; ftz-f1, ftz transcription factor 1.
(TIF)

S10 Fig Validation of Blimp-1 reagents. (A) Blimp-1 antibody staining in wild-type L3, 6-h, and 36-h wings corresponds to the gene expression changes of *Blimp-1*. (B) Expressing Blimp-1^RNAi^ in the posterior wings by *engrailed*-Gal4/Gal80^TS^ from 0 h APF reduces the level of Blimp-1 protein at 36-h wings. APF, after puparium formation; Gal80^TS^, temperature-sensitive Gal80; Blimp-1^RNAi^, RNA interference against Blimp-1.
(TIF)

S11 Fig Quantification of fluorescent images. This figure provides quantification for GFP reporters in Fig 2, fluorescent staining for chitin (Fig 5), and immunofluorescence for Blimp-1 (Fig 6). Images for GFP reporters were taken with the same exposure and gain at each stage (A). GFP intensity was measured from five to six comparable regions of two to five wings for each time point. All reporters exhibit significant changes in fluorescence intensity through one-way ANOVA test (Cpr51A *p*-value: <0.0001, stg *p*-value: <0.0001, e2f1 *p*-value: 0.0232, cycE *p*-value: 0.0005). (B) Chitin staining was compared between the dorsal and ventral epithelium for each wing, and the ratio was calculated with wild-type control wings set to 1. *N* = 3–5 wings for each genotype. Chitin signal is significantly affected by manipulating cell cycle exit (one-way ANOVA test, *p*-value: <0.0001). (C) Blimp-1 staining intensity was compared between posterior and anterior compartments of each wing, and the ratio (P:A) was calculated. *N* = 3–4 wings for each genotype. Bypassing cell cycle exit significantly delays the temporal regulation of Blimp-1 protein (36 h *p*-value: 0.0011; 41 h *p*-value: 0.0006, unpaired *t* test). The underlying data for this figure can be found within S7 Data. cycE, Cyclin E; Cpr51A, Cuticular protein 51A; E2F, E2F transcription factor; GFP, green fluorescent protein; P:A, posterior:anterior ratio; stg, string.
(TIF)

S1 Table FAIRE RPKM for high-confidence peaks in the wild-type time course and transgenic lines. FAIRE, formaldehyde-assisted isolation of regulatory elements; RPKM, reads per kilobase of transcript, per million mapped reads.
(XLSX)

S2 Table RPKM for the RNA-seq time course. RNA-seq, RNA sequencing; RPKM, reads per kilobase of transcript, per million mapped reads.
(XLSX)

S3 Table RNA-seq fold changes for all RNA-seq comparisons. RNA-seq, RNA sequencing.
(XLSX)

S1 Data Contains numerical data pertaining to Fig 1A–1D. (XLSX)

S2 Data Contains numerical data pertaining to Fig 2A, 2B and 2E. (XLSX)

S3 Data Contains numerical data pertaining to Fig 3E and 3D. (XLSX)

S4 Data Contains numerical data pertaining to Fig 4A and 4D. (XLSX)

S5 Data Contains numerical data pertaining to Fig 5A. (XLSX)

S6 Data Contains numerical data pertaining to Fig 6A. (XLSX)

S7 Data Contains numerical data pertaining to S1A and S1B, S2B–S2E, S6B, S8A and S8B, S9D and S11A–S11C Figs. (XLSX)

## Abbreviations

APC/C: anaphase-promoting complex/cyclosome
APF: after puparium formation
Cda5: Chitin deacetylase-like 5
cdc25c: cell division cycle 25c
Cdk: cyclin-dependent kinase
ChIP: chromatin immunoprecipitation
crol: crooked legs
CycD: Cyclin D
CycE: Cyclin E
Cpr51A: Cuticular protein 51A
DP: dimerization partner
DRE: DNA replication-related element
DREAM complex: dimerization partner, retinoblastoma-like E2F and MuvB complex
Dref: DRE-binding factor
E2F: E2F transcription factor
EcR: Ecdysone receptor
FAIRE: formaldehyde-assisted isolation of regulatory elements
FAIRE-seq: FAIRE sequencing
ftz-f1: ftz transcription factor 1
Gal80^TS^: temperature-sensitive Gal80
GO: Gene Ontology
H3K9Me3: H3K9 trimethylation
H3K27Me3: H3K27 trimethylation
HP1: heterochromatin protein 1
Hr3: Hormone receptor 3
hsflp: heatshock-flippase
LAD: lamina-associated domain
M1BP: Motif 1 binding protein
MEME: Multiple Em for Motif Elicitation
MMB: Myb-Muv B
ModENCODE: Model Organism Encyclopedia Of DNA Elements
Myb: Myb oncogene-like
ncRNA: noncoding RNA
orc6: origin recognition complex subunit 6
p55/CAF1: chromatin assembly factor 1, p55 subunit
PCNA: proliferating cell nuclear antigen
PRC1: Polycomb repressive complex 1
RB: Retinoblastoma-family protein
RNA-seq: RNA sequencing
rpkm: reads per kilobase of transcript, per million mapped reads
Starr-seq: self-transcribing active regulatory region sequencing; stg, string
Su(H): Suppressor of Hairless
TEM: transmission electron microscopy
TM6B: TM6B balancer
TSS: transcription start site
TwdlT: TweedleT
UAS: upstream activation sequence
ZP: zona pellucida
