# Supplementary figures and images for: Changes in chromatin accessibility ensure robust cell cycle exit in terminally differentiated cells
Source: PLoS Biol. 2019 Sep 3;17(9):e3000378. doi: 10.1371/journal.pbio.3000378 (PMC6743789; doi:10.1371/journal.pbio.3000378)

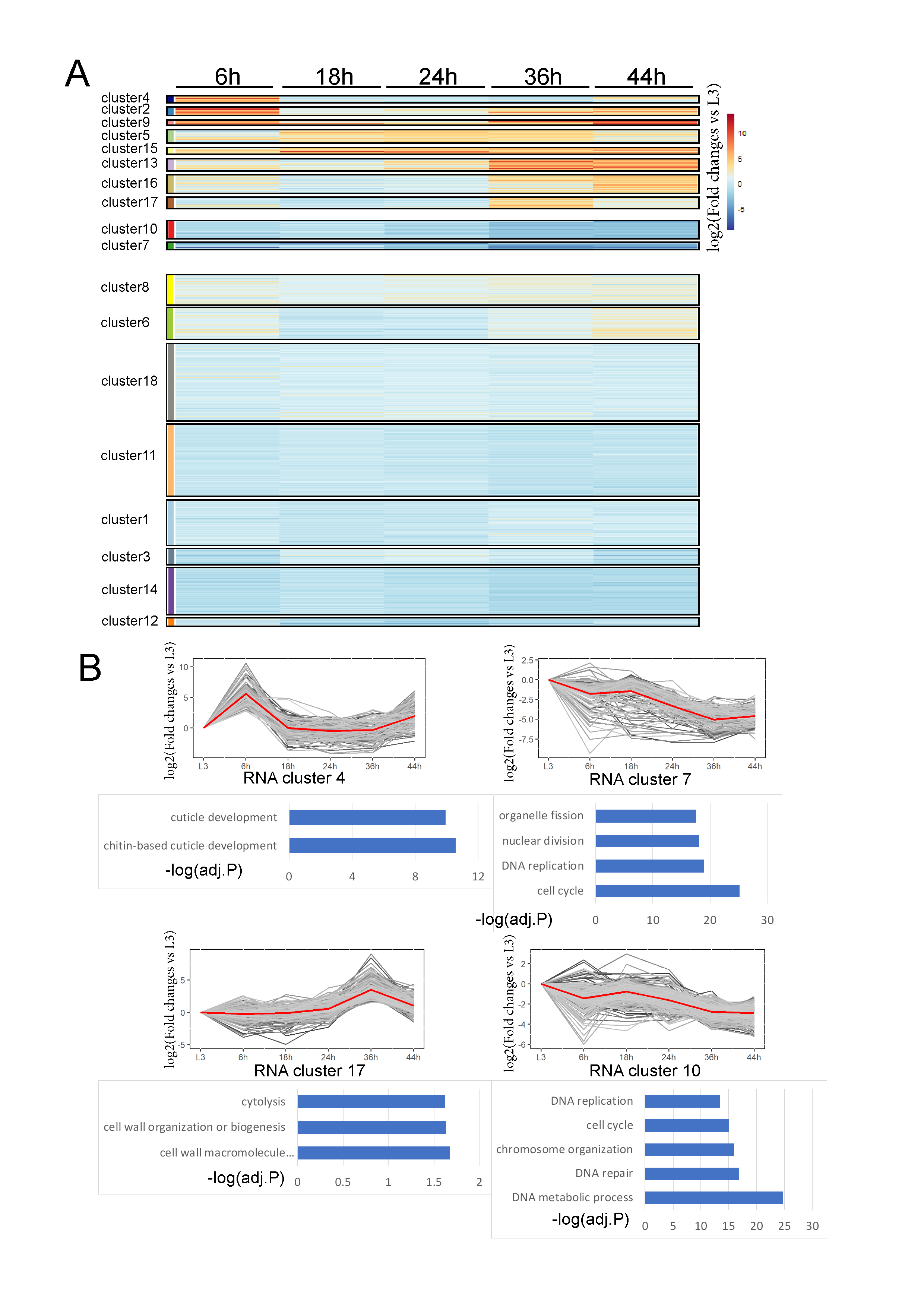

Supplement: S1 Fig — (A) The heatmap shows RNA log2 fold change (compared with L3) for the indicated stages. The pattern of RNA changes during metamorphosis is separated into 18 k-means clusters. (B) Line plots of the log2 fold change versus L3 for the indicated RNA clusters. Each gene is represented by a single gray line, and the average of all genes for the given cluster is plotted in red line. GO term enrichments are also shown along with their adjusted p-values. During metamorphosis, differentiation-related genes such as cuticle development are activated, whereas cell cycle genes are repressed. The underlying data for this figure can be found within S7 Data. GO, Gene Ontology. (TIF) [file pbio.3000378.s001.tif]

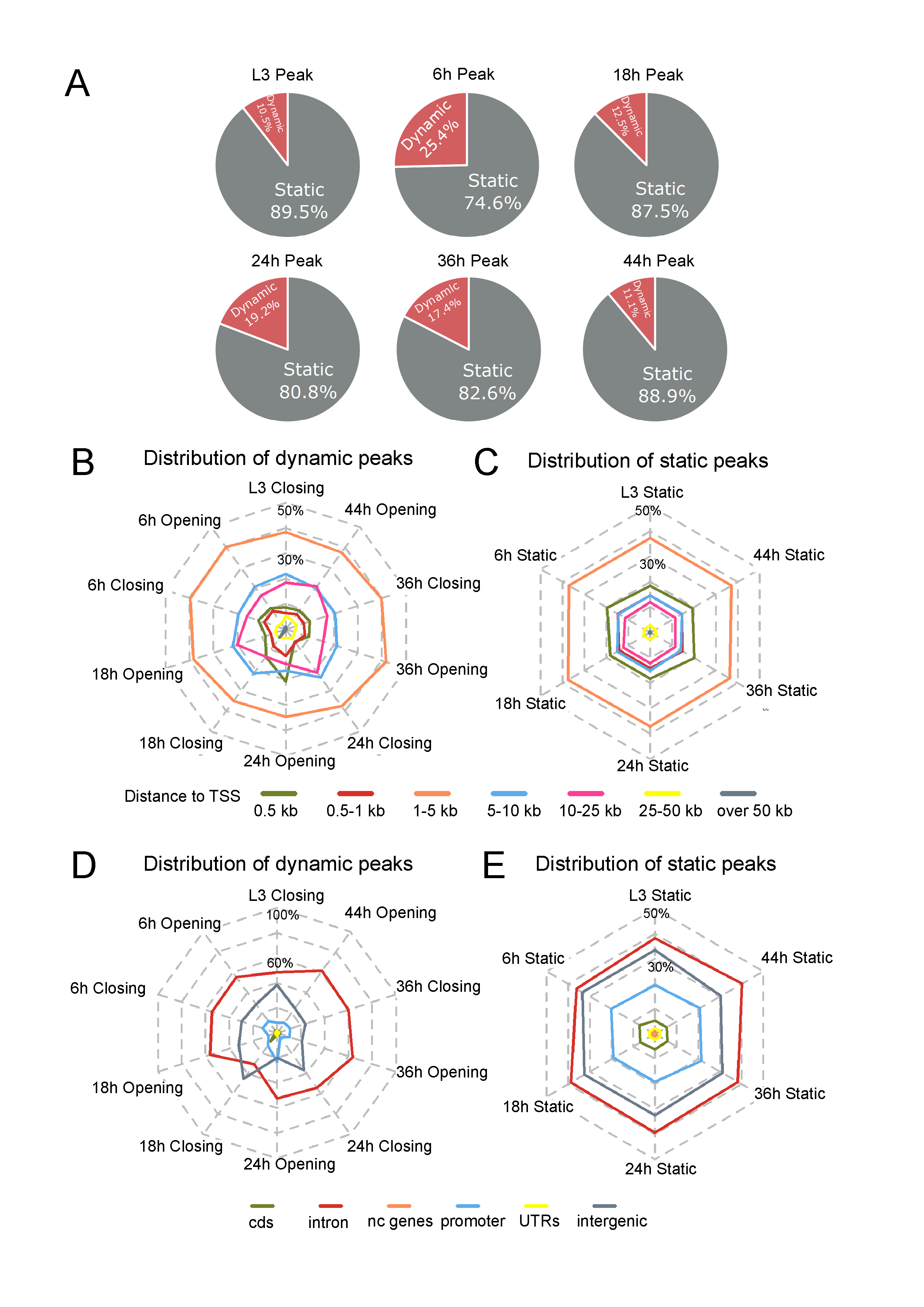

Supplement: S2 Fig — (A) Pie charts of the proportion of dynamic peaks and static peaks for each stage examined. Peaks without significant changes (<2-fold) between neighboring time points were defined as “static.” Peaks bearing changes >2-fold were defined as “dynamic.” (B, C) Radar charts display the distribution of indicated dynamic (B) and static (C) peak categories in different distances to TSS. (D, E) Radar charts display the distribution of indicated dynamic (D) and static (E) peak categories in cds, intron, nc genes, proximal promoter (−500 bp to 150 bp of TSS), UTRs, and intergenic regions. For dynamic peaks, “closing” is defined as peaks that decrease in accessibility by >2-fold compared with the previous stage; conversely, “opening” indicates peaks that increase in accessibility by >2-fold compared with the previous stage. The underlying data for this figure can be found within S7 Data. cds, coding sequence; nc gene, noncoding gene; TSS, transcription start site. (TIF) [file pbio.3000378.s002.tif]

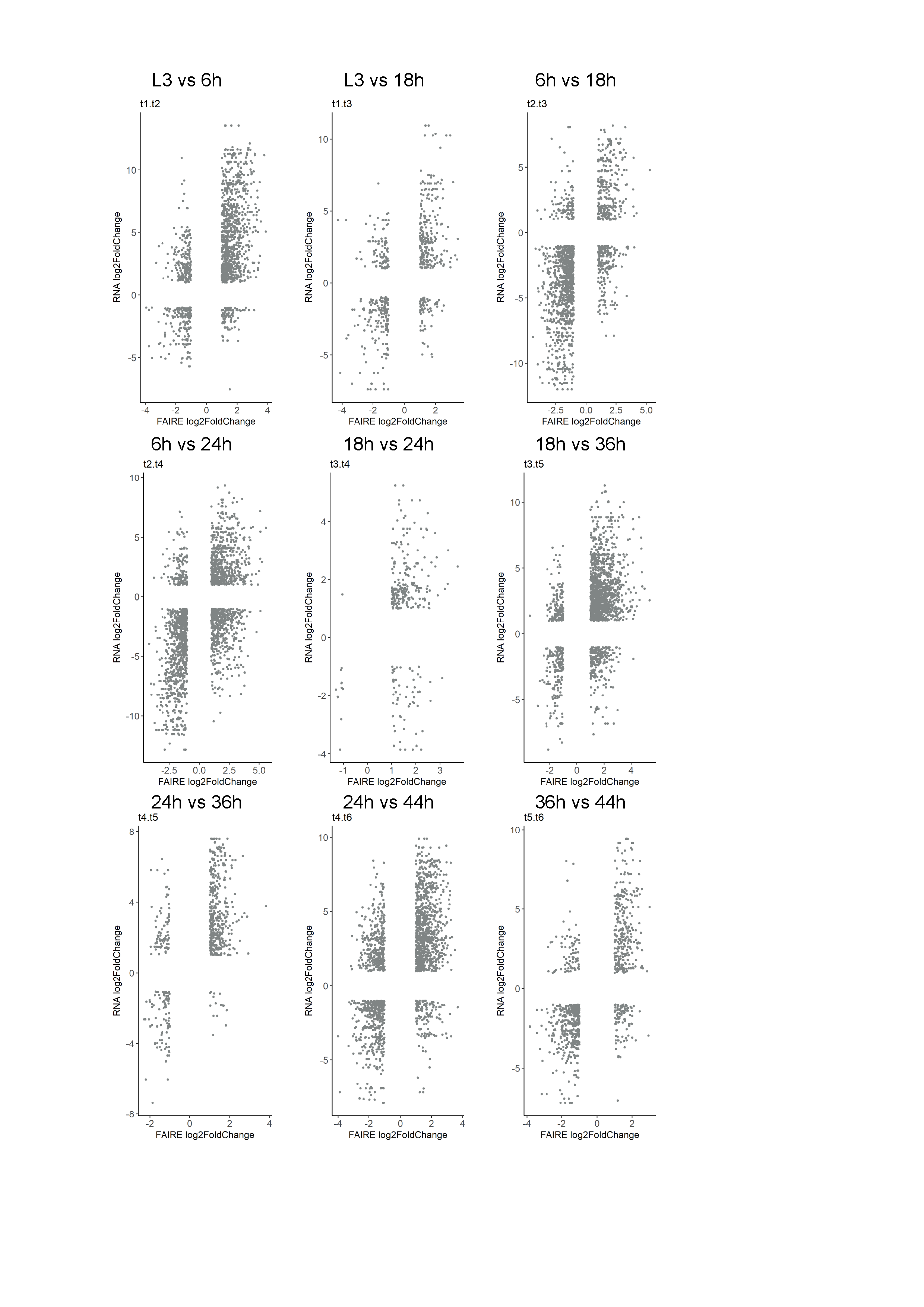

Supplement: S3 Fig — Scatterplots of FAIRE peaks and corresponding genes with significant changes between two sequential stages. Significance is defined by 2-fold changes and adjusted p-values less than 0.05. FAIRE, formaldehyde-assisted isolation of regulatory elements. (TIF) [file pbio.3000378.s003.tif]

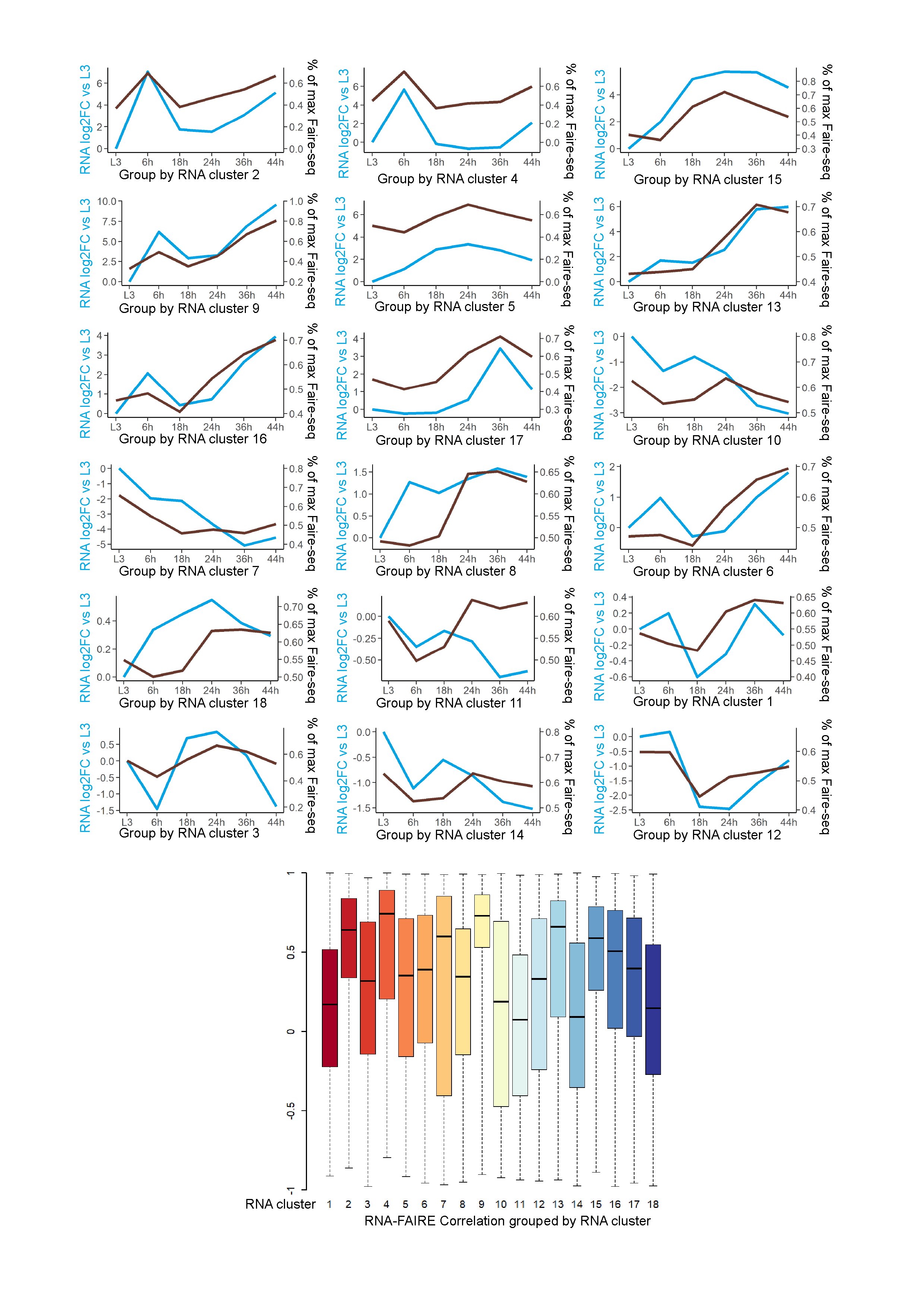

Supplement: S4 Fig — Trajectories of average changes between genes and their corresponding FAIRE peaks over the six stages for each of the 18 RNA clusters. Boxplot of the Pearson correlation coefficients between RNA and FAIRE for each RNA cluster is shown. The underlying data for this figure can be found within S7 Data. FAIRE, formaldehyde-assisted isolation of regulatory elements. (TIF) [file pbio.3000378.s004.tif]

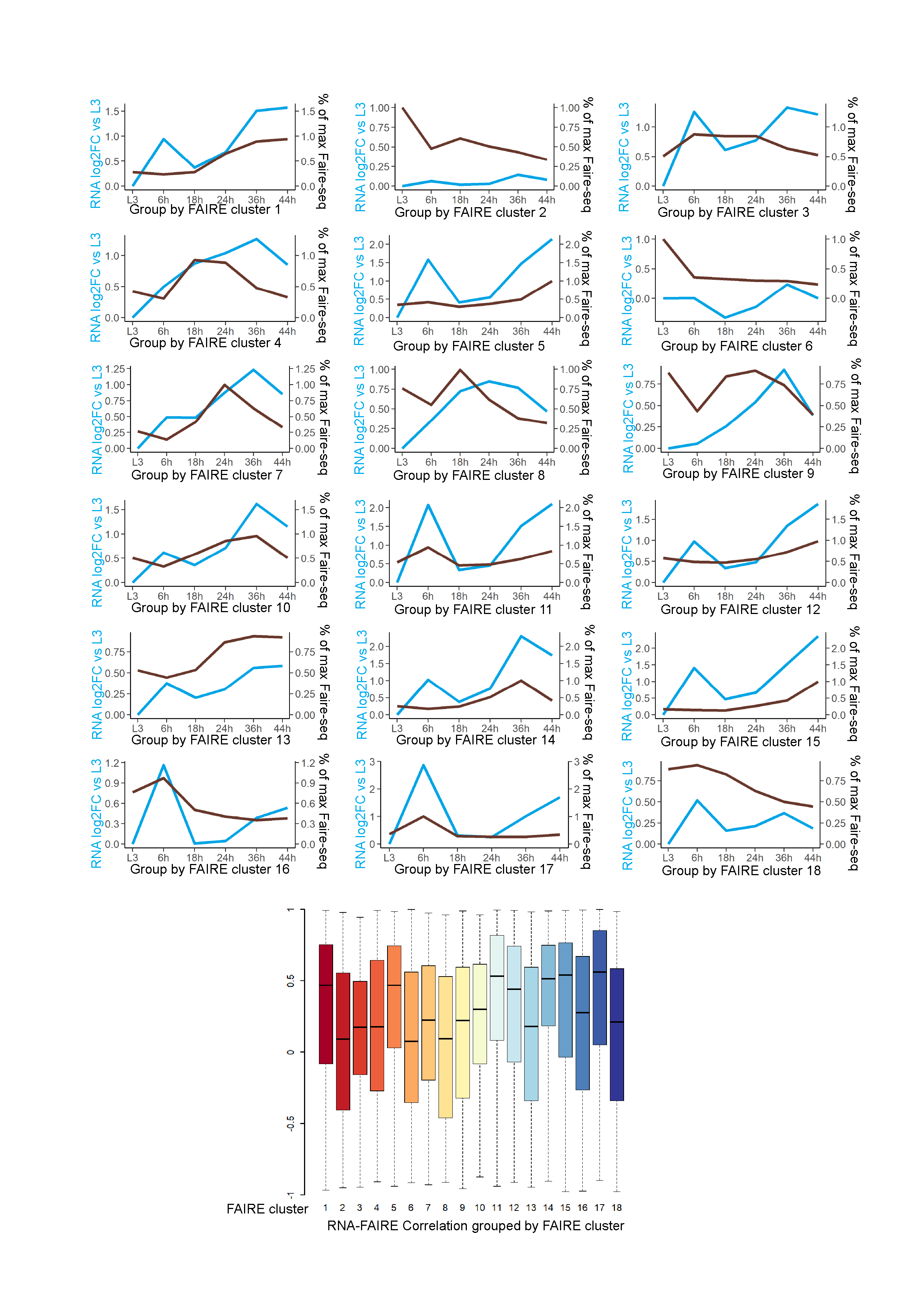

Supplement: S5 Fig — Trajectories of average changes between FAIRE peaks and their corresponding genes over the six stages for each of the 18 FAIRE clusters. Boxplot of the Pearson correlation coefficients between RNA and FAIRE for each FAIRE cluster is shown. The underlying data for this figure can be found within S7 Data. FAIRE, formaldehyde-assisted isolation of regulatory elements. (TIF) [file pbio.3000378.s005.tif]

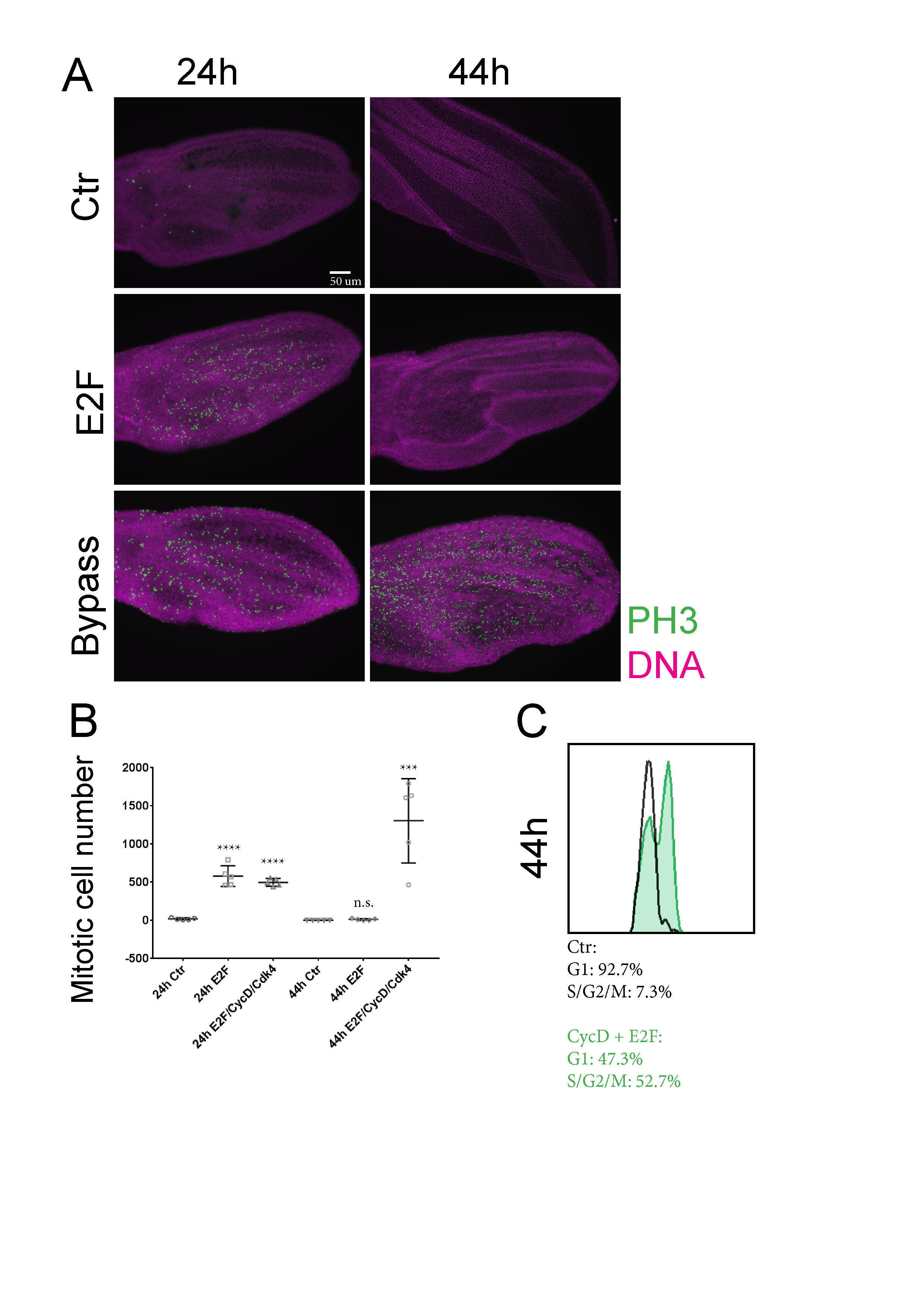

Supplement: S6 Fig — (A) E2F or E2F/CycD/Cdk4 (bypass) was overexpressed in the dorsal layer of wing epithelia under the control of Apterous-Gal4/Gal80ts from 12 h APF. The 24-h and 44-h wings were immunostained against PH3. (B) The number of PH3 spots of each wing is counted, and five wings for each genotype are quantified. (C) Cell cycle profile of the FAIRE samples that bypassed robust G0 by E2F/CycD/Cdk4 was examined by FACS. p-Values were determined by an unpaired t test; ****< 0.0001, ***< 0.001. The underlying data for this figure can be found within S7 Data. APF, after puparium formation; Cdk, cyclin-dependent kinase; CycD, Cyclin D; E2F, E2F transcription factor; FACS, Fluorescence-activated cell sorting; FAIRE, formaldehyde-assisted isolation of regulatory elements; Gal80TS, temperature-sensitive Gal80; PH3, phosphohistone H3. (TIF) [file pbio.3000378.s006.tif]

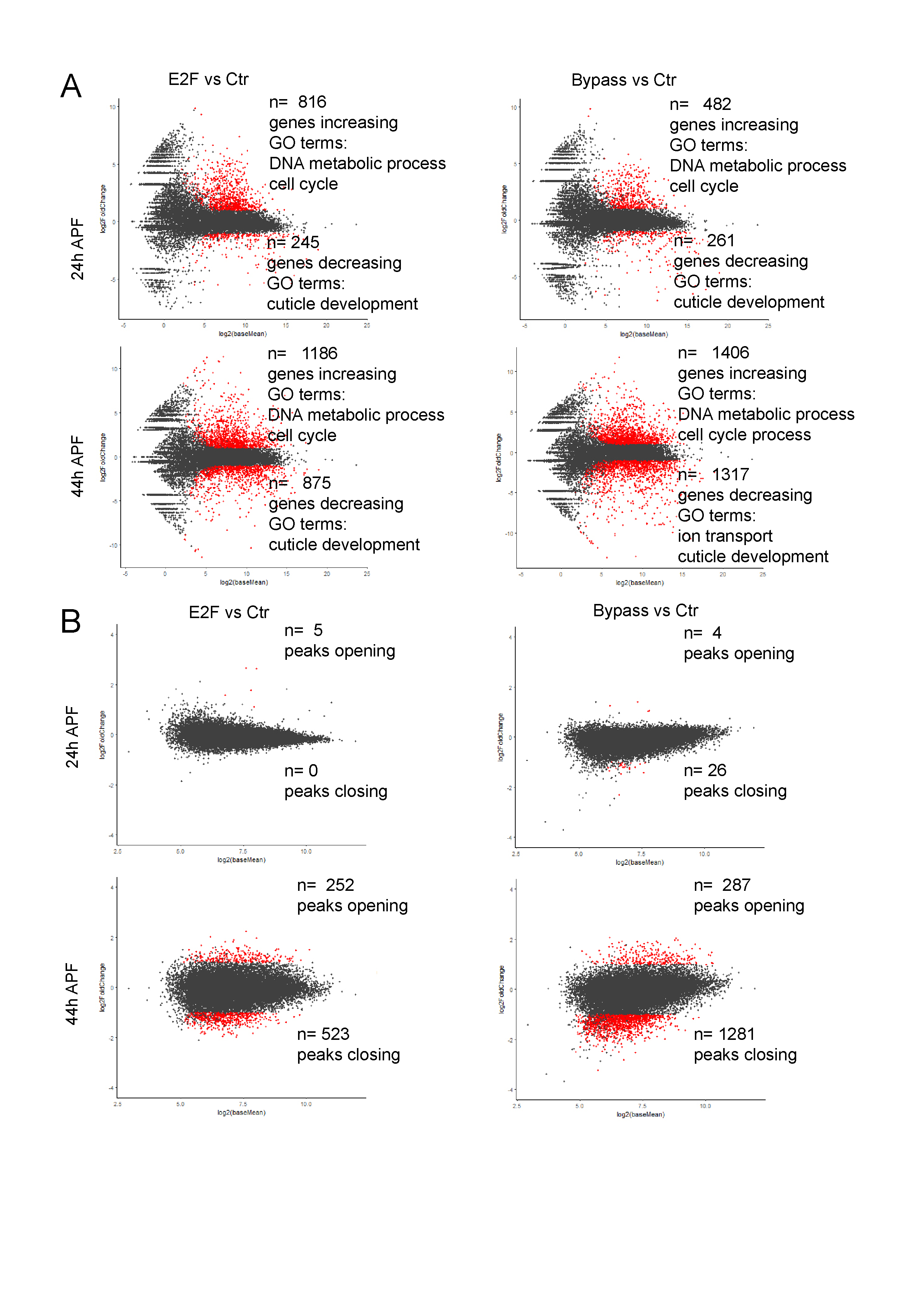

Supplement: S7 Fig — MA plots of RNA (A) and FAIRE (B) changes of 24- and 44-h wings compared with control. Genes and peaks that are significant in changes with 2-fold difference and adjusted p-value less than 0.05 are labeled in red. Cdk, cyclin-dependent kinase; CycD, Cyclin D; E2F, E2F transcription factor; FAIRE, formaldehyde-assisted isolation of regulatory elements; FAIRE-seq, FAIRE sequencing; MA plot, scatter plot onto M (log ratio) and A (mean average) scales; RNA-seq, RNA sequencing. (TIF) [file pbio.3000378.s007.tif]

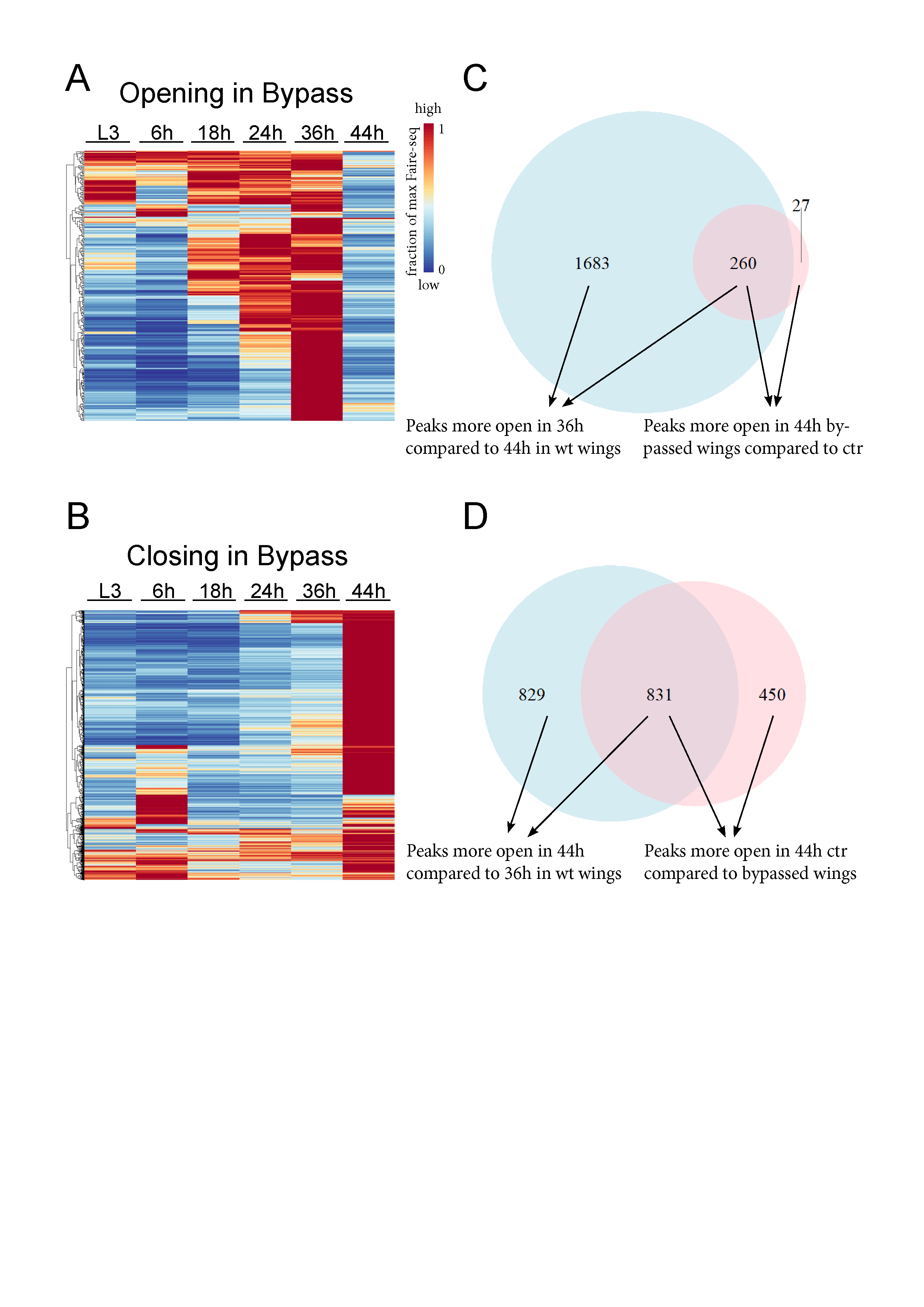

Supplement: S8 Fig — (A, B) The heatmap shows the temporal dynamics during normal development for the peaks that are more accessible or less accessible at 44-h wings expressing E2F/CycD/Cdk4, plotted as a fraction of the maximum FAIRE rpkm value. Compromising G0 leads to the failure of proper closing of 36-h peaks as well as delayed opening of 44-h peaks. (C) Overlap between peaks that normally open at 36 h in wild-type and peaks more accessible at 44 h bypassed wings. (D) Overlap between peaks that normally open at 44 h in wild-type and peaks less accessible at 44 h bypassed wings. The underlying data for this figure can be found within S7 Data. Cdk, cyclin-dependent kinase; CycD, Cyclin D; E2F, E2F transcription factor; FAIRE, formaldehyde-assisted isolation of regulatory elements; rpkm, reads per kilobase of transcript, per million mapped reads. (TIF) [file pbio.3000378.s008.tif]

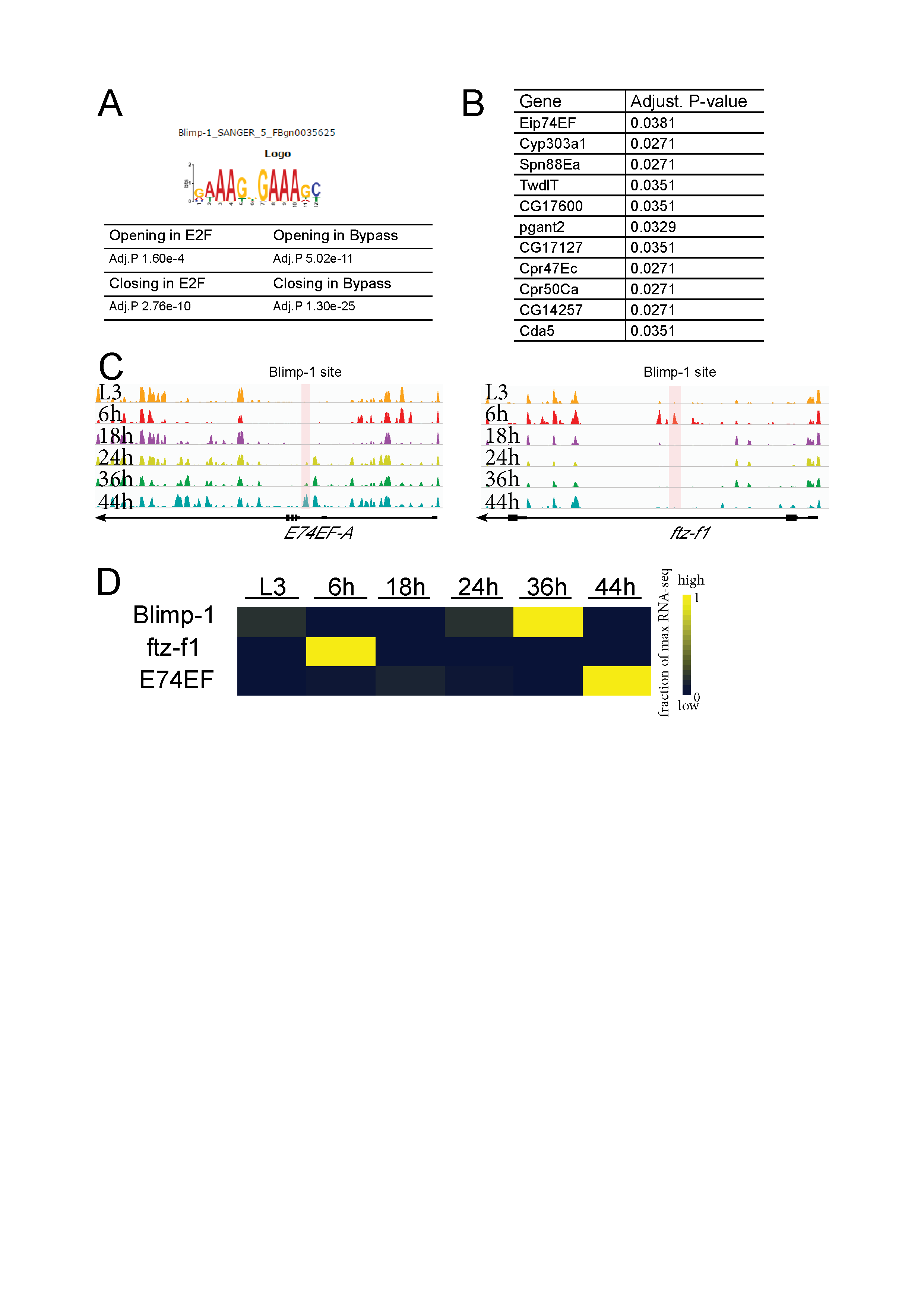

Supplement: S9 Fig — (A) The Blimp-1 motif is enriched in the dynamic peaks disrupted by E2F or bypass found by AME analysis. (B) A list of genes containing peaks that fail to open at 44 h with high-scoring Blimp-1 binding sites. (C) Chromatin accessibility changes at E74EF and ftz-f1 loci with Blimp-1 binding sites are shown. (D) Expression changes of Blimp-1, ftz-f1, and E74EF during normal development. The underlying data for this figure can be found within S7 Data. AME, Analysis of Motif Enrichment; E2F, E2F transcription factor; ftz-f1, ftz transcription factor 1. (TIF) [file pbio.3000378.s009.tif]

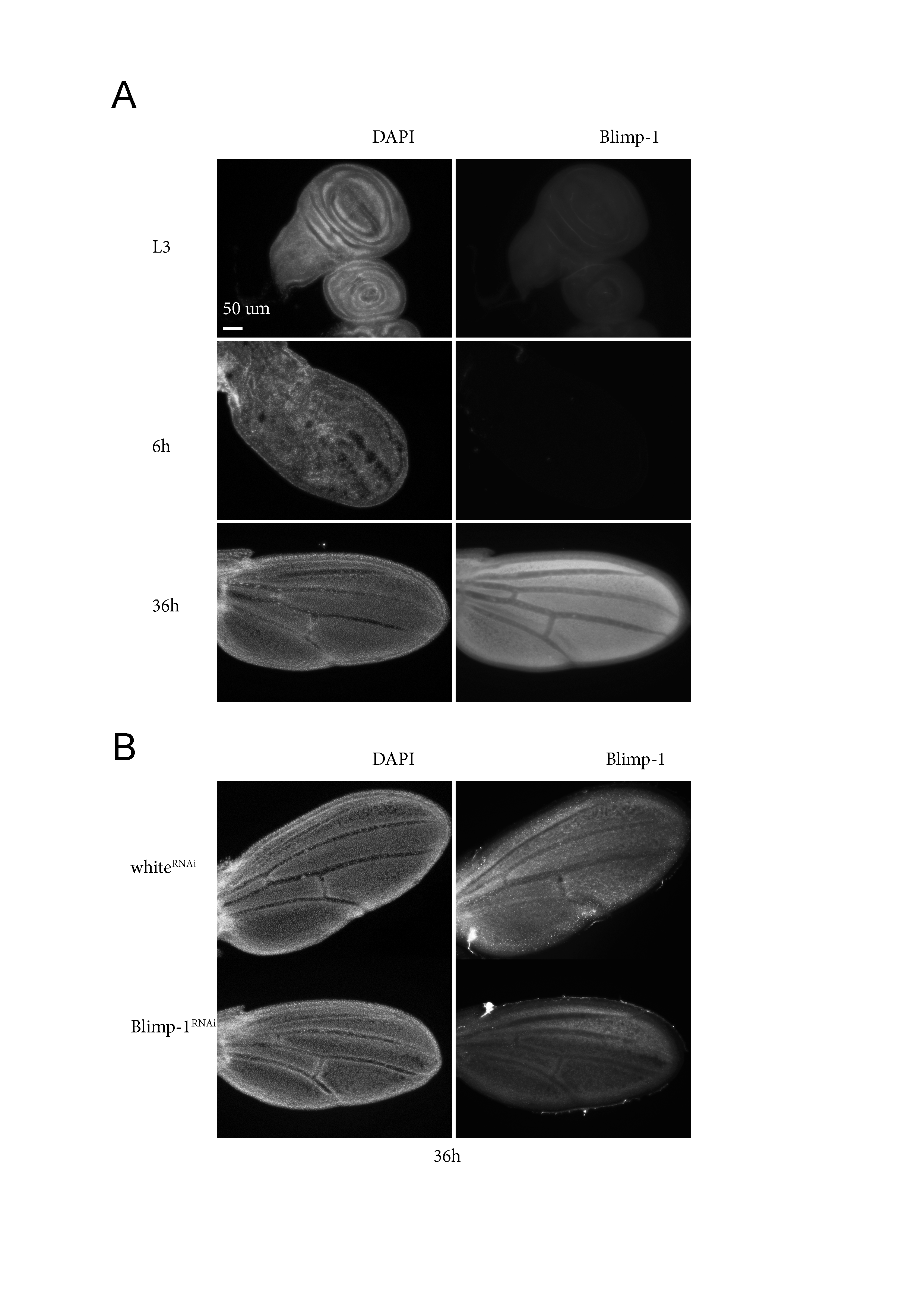

Supplement: S10 Fig — (A) Blimp-1 antibody staining in wild-type L3, 6-h, and 36-h wings corresponds to the gene expression changes of Blimp-1. (B) Expressing Blimp-1RNAi in the posterior wings by engrailed-Gal4/Gal80TS from 0 h APF reduces the level of Blimp-1 protein at 36-h wings. APF, after puparium formation; Gal80TS, temperature-sensitive Gal80; Blimp-1RNAi, RNA interference against Blimp-1. (TIF) [file pbio.3000378.s010.tif]

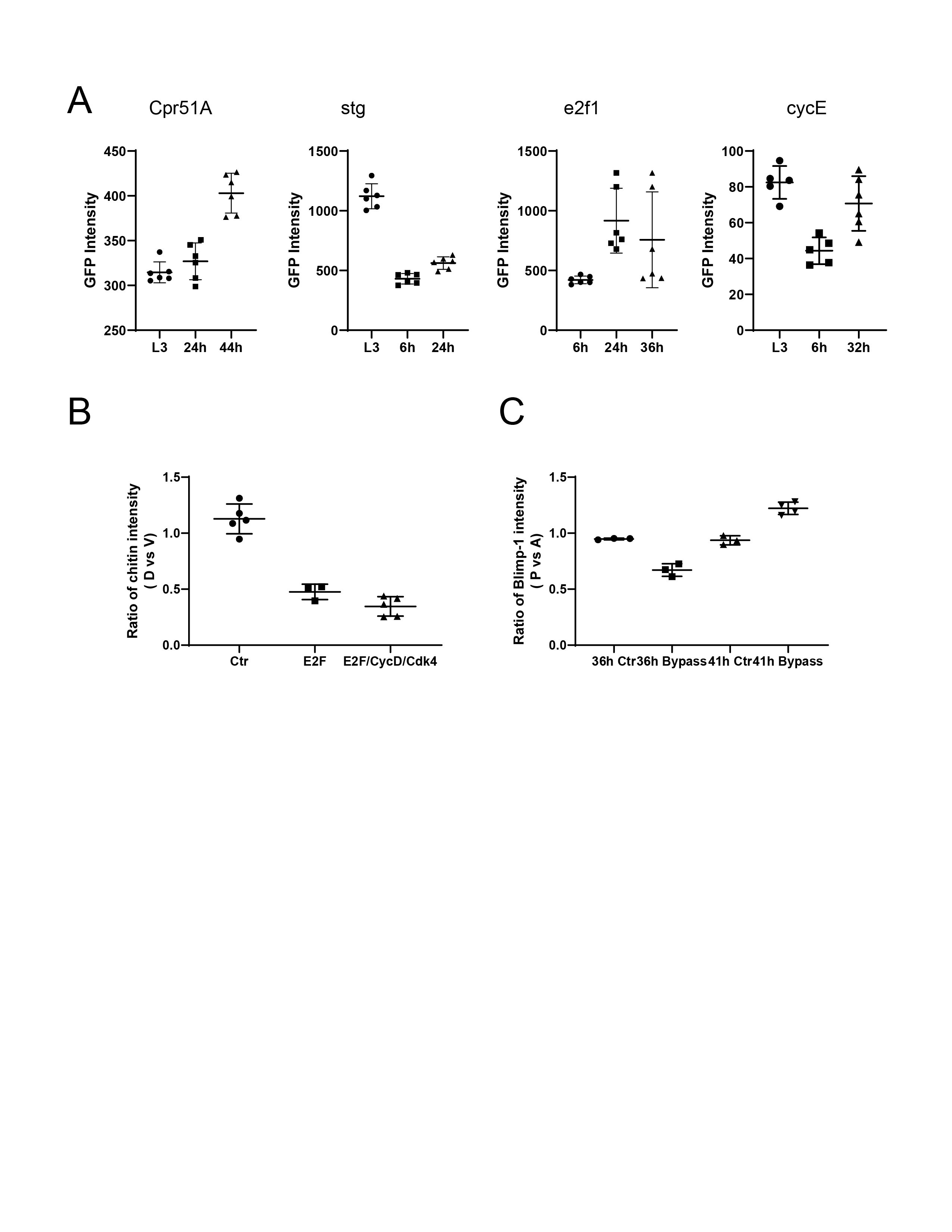

Supplement: S11 Fig — This figure provides quantification for GFP reporters in Fig 2, fluorescent staining for chitin (Fig 5), and immunofluorescence for Blimp-1 (Fig 6). Images for GFP reporters were taken with the same exposure and gain at each stage (A). GFP intensity was measured from five to six comparable regions of two to five wings for each time point. All reporters exhibit significant changes in fluorescence intensity through one-way ANOVA test (Cpr51A p-value: <0.0001, stg p-value: <0.0001, e2f1 p-value: 0.0232, cycE p-value: 0.0005). (B) Chitin staining was compared between the dorsal and ventral epithelium for each wing, and the ratio was calculated with wild-type control wings set to 1. N = 3–5 wings for each genotype. Chitin signal is significantly affected by manipulating cell cycle exit (one-way ANOVA test, p-value: <0.0001). (C) Blimp-1 staining intensity was compared between posterior and anterior compartments of each wing, and the ratio (P:A) was calculated. N = 3–4 wings for each genotype. Bypassing cell cycle exit significantly delays the temporal regulation of Blimp-1 protein (36 h p-value: 0.0011; 41 h p-value: 0.0006, unpaired t test). The underlying data for this figure can be found within S7 Data. cycE, Cyclin E; Cpr51A, Cuticular protein 51A; E2F, E2F transcription factor; GFP, green fluorescent protein; P:A, posterior:anterior ratio; stg, string. (TIF) [file pbio.3000378.s011.tif]
